# Long non-coding RNA HOXA11-AS knockout inhibits proliferation and overcomes drug resistance in ovarian cancer

**DOI:** 10.1080/21655979.2022.2086377

**Published:** 2022-06-15

**Authors:** Yuwei Chen, Zhaolei Cui, Qiaoling Wu, Huihui Wang, Hongmei Xia, Yang Sun

**Affiliations:** a Department of Gynecology, Fujian Medical University Cancer Hospital, Fujian Cancer Hospital, Fuzhou, China; b Laboratory of Biochemistry and Molecular Biology Research, Department of Clinical Laboratory, Fujian Medical University Cancer Hospital, Fujian Cancer Hospital, Fuzhou, China; c Department of Gynecology, Fujian Cancer Hospital, Fuzhou, China

**Keywords:** Ovarian cancer, HOXA11-AS, autophagy, cisplatin resistance, lncRNA

## Abstract

In ovarian carcinogenesis and progression, long non-coding RNAs (lncRNAs) have been shown to have a role, although the underlying processes remain a mystery. By modulating the degree of autophagy in ovarian cancer cells, we sought to learn more about the function lncRNA HOXA11-AS plays in the development of ovarian cancer. The expression of HOXA11-AS in ovarian normal cells and ovarian cancer cell lines was measured using R package and qRT-PCR. Ovarian cancer cells expressed HOXA11-AS substantially higher than normal cells, while cisplatin-resistant cells expressed HOXA11-AS significantly higher than ovarian cancer cells. Next, we studied the prognostic data of HOXA11-AS in ovarian cancer in the Tissue Cancer Genome Atlas (TCGA). In the next step, lentiviral transfection of ovarian cancer cells A2780, OVCAR3, and A2780/DDP (cisplatin-resistant) were performed, and HOXA11-AS knockdown was found to significantly inhibit cell viability, migration, and invasion of A2780 and OVCAR3 cells, and promote apoptosis by CCK-8 assay, transwell assay, cell cycle, and apoptosis assay, and promoted the sensitivity of A2780/DDP cells to cisplatin. It has been shown by the western blot test that HOXA11-AS knockdown increases the amount of cellular autophagy in cells. In contrast, adding the autophagy inhibitor 3-methyladenine (3-MA) to HOXA11-AS cells knocked down in vivo reduced its anti-tumor properties. As a whole, this study found that HOXA11-AS knockdown increased the expression of autophagy-related proteins and improved cisplatin sensitivity, decreased ovarian cancer cell proliferation, and promoted cell apoptosis. This study provides new insights into the role of HOXA11-AS in ovarian cancer regulation.

## Graphical abstract

**Figure:**
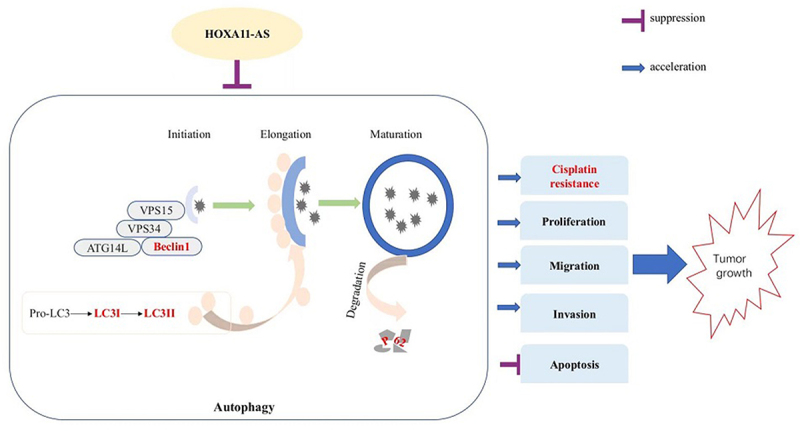

## Research Highlights:

- To explore the mechanism of development and drug resistance of ovarian cancer;
- To study the association between lncRNA HOXA11-AS and autophagy;
- To uncover mechanisms for HOXA11-AS’s role in ovarian cancer progression using bioinformatics analysis and cell experiments.

## 1. Introduction

Ovarian cancer is the most lethal malignant tumor of female genitalia with increasing morbidity and mortality [1]. Although the combined application of cytoreductive surgery and neoadjuvant chemotherapy has increased the survival rate of some patients, 5-year survivors only account for 49% of this patient group [2] mainly because most of them have developed the advanced-stage disease at diagnosis [3]. In addition, they are prone to relapse after treatment. The high recurrence rate and short survival rate of individuals with ovarian cancer are partly due to the rapid acquisition of chemoresistance [4]. Therefore, it is of great significance to further study the pathogenesis and drug resistance mechanism of ovarian cancer and provide therapeutic targets and effective treatment strategies for ovarian cancer.

As a source of energy to maintain a highly integrated intracellular homeostasis, autophagy is the recycling of intracellular material [5]. This is vital for preserving the body’s equilibrium. Consequently, an imbalance in autophagy is connected with several illnesses, including cancer, neurological disorders, and cardiovascular disorders [6]. Tumor suppression relies heavily on autophagy-dependent cell death. For the prevention of carcinogenesis, proliferation, invasion, and metastatic spread, autophagy can act as a tumor-suppressive mechanism by regulating intracellular protein and organelle quality, preserving genomic integrity, and suppressing oncogenic protein aggregate formation [7]. Down-regulation of autophagy-related genes in tumor tissues, or changes in the genes themselves, can increase the likelihood of tumor formation [8]. By mining the Cancer Genome Atlas (TCGA) and Gene Expression Omnibus (GEO) data, it was found that autophagy-related genes can predict the prognosis of ovarian cancer patients [9,10]. This confirms that autophagy plays a crucial role in the development of ovarian cancer.

Long non-coding RNAs (lncRNAs), a class of RNA transcripts with a length greater than 200 nucleotides, have been shown to participate in dose compensation effect, epigenetic regulation, cell cycle regulation, and cell differentiation regulation [11,12]. In recent years, aberrant lncRNA expression has been found to involve the onset and progression of numerous tumors. Among others, lncRNA HOTAIR, H19, MALAT1, and HOST2 can be used as potential therapeutic targets for ovarian cancer and are closely related to tumorigenesis and metastasis in ovarian cancer [13–16]. An old and evolutionary conserved collection of genes, the HOX (homeobox) family, regulates essential genetic developmental processes [17]. Genes of the HOX family may function as tumor suppressors or oncogenes, and their primary mechanism of action is the activation or repression of target genes through transcription [18]. In the regulation of autophagy, HOX gene can act as a central regulatory factor of autophagy and play a role by regulating autophagy-related genes (ATGs) [19]. An autophagy inhibitor, the Drosophila HOX protein, has been discovered to be an effective inhibitor of autophagy in both development and starvation [20]. lncRNA HOXA11-AS, a member of the HOX gene family, is characterized by highly conserved homology domains, in the human genome [21]. HOXA11-AS regulates the progression of several tumors. HOXA11-AS overexpression has been shown to fuel cell proliferation and migration in glioma cells by constructing a ceRNA network with miR-214-3p [22]. HOXA11-AS may also predict a poor prognosis in breast cancer, hepatocellular carcinoma, uveal melanoma, and osteosarcoma [23–26]. In addition, HOXA11-AS was found to promote chemotherapy resistance. For example, Wang et al. found that LncRNA HOXA11-As promoted the proliferation and cisplatin resistance of oral squamous cell carcinoma by inhibiting mir-214-3p expression [27]. However, there are few studies on HOXA11-AS in ovarian cancer. In 2017, it was found that the expression of HOXA11-AS in serous ovarian cancer was significantly higher than that in normal ovarian tissue, accompanied by higher histological grade and cancer antigen 125 (CA125) level [28]. But the exact roles of HOXA11-AS in ovarian cancer cell behavior remains unclear. At present, there is no relevant research on how HOXA11-AS regulates intracellular autophagy and cisplatin resistance in ovarian cancer.

This study aims to investigate the expression and function of HOXA11-AS in ovarian cancer cell lines. In addition, the potential mechanism of HOXA11-AS on ovarian cancer cell progression and chemotherapy resistance was also studied. Our results showed that HOXA11-AS could promote the proliferation and migration of ovarian cancer cells by inhibiting intracellular autophagy levels, and promote cisplatin resistance of ovarian cancer cells, so as to provide the evidence that HOXA11-AS may be a therapeutic target for ovarian cancer.

## 2. Materials and methods

### 2.1 Cell culture and transfection

We selected three human ovarian cancer cell lines A2780, SKOV3, and OVCAR3, a cisplatin-resistant ovarian cancer cell line A2780/DDP, and immortalized human ovarian surface epithelial (IOSE-80) cells for experiments (BeNa Culture Collection, China). Cells were maintained in RPMI-1640 (HyClone, USA) supplemented with 10% fetal bovine serum (FBS) (Gibco, Grand Island, NY) at 37°C with 5% CO_2_. To maintain the cellular drug resistance, A2780/DDP cells were cultivated in RPMI-1640 containing 1 μg/mL of Cisplatin. After the cells had grown to the logarithmic phase, A2780, OVCAR3, and A2780/DDP cells (3 × 10^5^ cells/well) were grown in 6-well plates overnight at 37°C, reaching a density of 60%–70%. Cris-HOXA11-AS (Multiplicity of Infection = 100) and con-HOXA11-AS were transfected into cells. Lentivirus transfection efficiency of more than 80% was considered successful. After 24 h(hour) of transfection, the medium was replaced to remove lentivirus particles. After 48 h, Purinomycin resistant cells were amplified in 20 μg/ml puromycin medium for 2 days. cris-HOXA11-AS and con-HOXA11-AS lentivirus were purchased from HanBio Technology (Shanghai, China). cris-HOX11-AS-as lentivirus adopts CRISPR-Cas9 system mediated by lentivirus [29].

### 2.2 qRT-PCR assay

A2780/DDP, A2780, SKOV3, OVCAR3, and IOSE-80 cells were homogenated to isolate total RNA by using the MIX method (Vazyme Biotech Co, Ltd), and a reverse transcription step was done using the Hiscript III qRTSuperMix reverse transcription kit (Vazyme Biotech Co, Ltd). This cDNA was then used as a template, and a fluorescent qRT-PCR detection kit (ChamQ Universal SYBR qPCR Master Mix, Vazyme Biotech Co, Ltd) was used to determine the expression of lncRNA HOXA11-AS [30]. The upstream primer sequence of lncRNA HOXA11-AS was 5’-CACGGTGACTTGATTACACTCTC-3’, whereas its downstream primer sequence was 5’-AGGTAGGCAGGGAAGATGAG −3’. The upstream and downstream primer sequences of GAPDH (internal reference) were 5’-CAGCCTCAAGATCATCAGCA −3’ and 5’-TGTGGTCATGAGTCCTTCCA −3’, respectively. The primers were synthesized by Shangya Biotechnology (Fuzhou). The PCR reaction conditions were as follows: 95°C for 30s; 95°C for 10s; and 60°C for 30s for 40 cycles. LncRNA HOXA11-AS expression was calculated as a fold change of the 2^−ΔΔCt^ value and normalized to GAPDH expression.

### 2.3 Cell counting kit (CCK)-8 assay

CCK-8 assays (Beyotime, Nanjing, China) were performed to determine cell growth. The transfected cells were inoculated in a 96-well plate at 5 × 10^3^ cells for each well, and the medium volume per well was 100 μL. After culturing of OVCAR3 and A2780 cells in an incubator for 0, 24, 48, and 72 h, 10 μL of CCK-8 agent per well test solution was added. Cells were incubated at 37°C with 5% CO_2_ for 1 h. For drug sensitivity experiments, A2780/DDP cells were exposed to 0,1, 5, 10, 20 and 40 μg/mL of cisplatin for 24 h before 1-h incubation of the CCK-8 agent at 37°C with 5% CO_2_. The optical density at 450 nm was measured with a microplate reader (Molecular Devices, USA) [31]. In the rescue experiment, the cells in cris-HOXA11-AS-1 group were pretreated with 5mM3-methyladenine per well for 4 hours.

### 2.4 Cell cycle assay

The Meilun Cell Cycle Kit was used to detect cell cycle (meilunbio, Dalian, China). Cells grown at the logarithmic phase (60% density) were transferred to a 6-well plate and cultured in an incubator for 24 h, followed by enzymatic digestion (trypsin), centrifugation at 800 rpm for 5 min to collect cell pellets. They were washed in precooled phosphate-buffered saline (PBS) and 75% ethanol and purified by centrifugation at 1,500 rpm for 5 min. Samples were treated with a mixture (500ul) of RNaseA, propidium iodide (PI), and staining buffer (2:5:100) in the dark for 30 min at 37°C [32]. A flow cytometer was used, as per standard procedures, for detection. Cell cycle fractions were analyzed with ModFit.

### 2.5 Transwell assay

The transfected OVCAR3 and A2780 cells (1 × 10^4^ cells/100 μL) were added to the upper chamber in a serum-free medium. Medium supplemented with 15% FBS was placed in the lower chamber. A matching 24-well plate with the chamber was placed in the incubator for 24 h. They were fixed with paraformaldehyde for 15 min and stained with crystal violet at room temperature for 25 min after 24 hours of incubation with 4% crystal violet. Cells migrated to the lower chamber were stained with crystal violet, photographed, and counted under a microscope (Leica DMIL, LED, Germany). For the invasion experiment, cells (4 × 10^4^ cells/100 μL) were cultured on a Matrigel-coated upper chamber (8-µm pore size inserts; Corning, Inc.) [33]. The remaining steps are similar to the previous steps.

### 2.6 Apoptosis assay

In the present study, the Annexin V/PE cell apoptosis kit (Keygene Biotech, Nanjing, China) was used to evaluate the apoptosis of OVCAR3 and A2780 cells. The cells were digested into a suspension with trypsin without EDTA (ethylenediamine tetraacetic acid), followed by a 5-min centrifugation at 1000 rpm. Cell pellets were washed twice with precooled PBS before adding 750 μL of binding buffer working solution to resuspend the cells. Cells were stained with Annexin V (Annexin V(FITC)) (5ul) and propidium iodide(PI) (5ul) in the dark for 15 min (minute) at room temperature [34]. Following the staining incubation, 400ul 1× Binding Buffer was added to each tube. We employed flow cytometry to count apoptotic cells.

### 2.7 Western blot

As described by Zhong, et al [34]. Radio immunoprecipitation (RIPA) buffer (Keygen Biotech) and phenylmethylsulfonyl fluoride were mixed at a 100:1 ratio. The protein from each group of cultured cells was extracted according to the instructions. After centrifugation at 1000 × g for 20 min, the supernatant was collected, and the bicinchoninic acid assay method was utilized for quantification of the total protein (Thermo Fisher Scientific, USA). An equal amount of protein (30 μg) was loaded in each lane. The concentrated gel was electrophoresed at 80 V for 40 min, and the separation gel was electrophoresed at 100 V for 2 h. Approximately 200 mA of current was used to transfer the polyvinylidene fluoride (PVDF) membranes (Millipore, USA), followed by the blocking step (5% skimmed dry milk). Membranes were incubated with anti-P62 (1:1000), Beclin1 (1:1000), LC3 I/II (1:1000), and β-actin (1: 5000) primary antibodies overnight at 4°C. The next day, add corresponding secondary antibodies were incubated for 2 h, and the luminescent solution was exposed and developed (Thermo Fisher Scientific). Protein expression levels were quantified with ImageJ software and normalized to the loading control β-actin.

### 2.8 Downloading of information and differential expression analysis

There were 53 ovarian cancer samples and 10 normal ovarian tissues on the GSE18520 (GPL570) microarray. 5 ovarian cancer cisplatin-sensitive cells and 5 ovarian cancer cisplatin-resistant cells were present on the GSE15372 (GPL570) microarray. On both microarrays, differential expression analysis between groups was conducted using the limma package [35]. According to the criterion |log2 (Fold change)| >1 and P.val 0.05, differentially expressed lncRNAs were chosen. The ggplot2 software was used to display the findings.

### 2.9 Statistical analysis

All statistical analyses were performed on GraphPad prism 8.0 and R software (version 4.10). Continuous variables were presented in mean ± standard deviation (SD). The mean difference between the two groups was tested using a t-test. The significance level was set at *P* < 0.05.

## 3. Results

In this work, we investigated the effect of lncRNA HOXA11-AS on the proliferation and cisplatin resistance of ovarian cancer cells. Using R package and qRT-PCR, we analyzed the expression levels of lncRNA HOXA11-AS in ovarian cancer cell lines. We investigated the biological effects of lncRNA HOXA11-AS knockdown on the proliferation, metastasis, and cisplatin resistance of ovarian cancer cells. We observed that knockdown of lncRNAHOXA11-AS increases the amount of autophagy in ovarian cancer cells, and the addition of an autophagy inhibitor partly restored the reduced cellular activity in knockdown lncRNA HOXA11-AS cells. The following are the study’s conclusions.

### 3.1 HOXA11-AS is elevated in ovarian cancer

**Figure 1.**
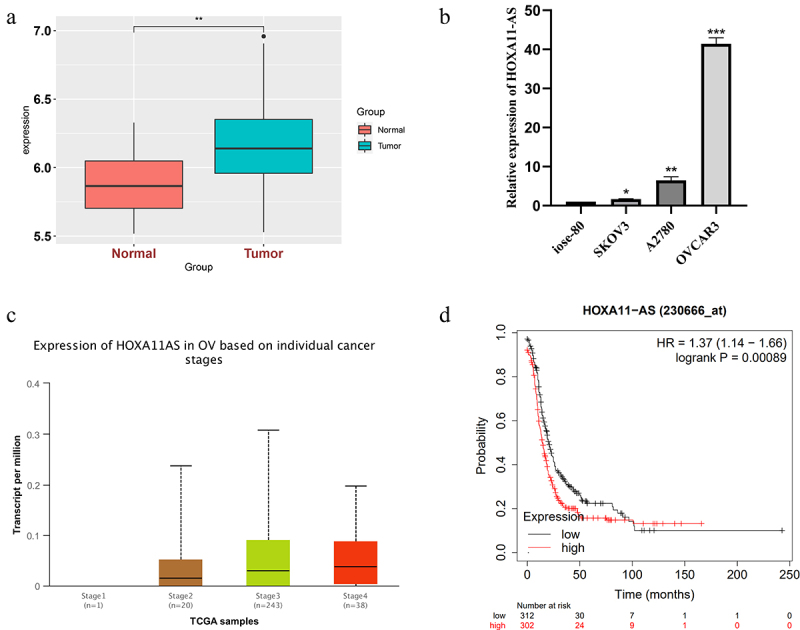
Elevation of HOXA11-AS expression in ovarian cancer tissues and cells. (a) lncRNA HOXA11-AS is markedly elevated in GSE18520 dataset ovarian cancer tissue samples. (b) qRT-PCR was used to quantitate HOXA11-AS expression in SKOV3, OVCAR3, A2780, and IOSE-80 cells. (c)The Tissue Cancer Genomic Atlas (TCGA) visualization database UALCAN exhibits HOXA11-AS expression under different stages of ovarian cancer development. (d) Kaplan–Meier curve illustrating the influence of differing HOXA11-AS upregulation on ovarian cancer prognosis. * P < 0.05, ** P < 0.01 and ** P < 0.01 vs. respective control, and the results are from three independent repeated experiments.

In the GSE18520 dataset, HOXA11-AS was pronouncedly elevated in ovarian cancer tissue samples (Figure 1(a)). Subsequently, qRT-PCR was used to quantitate HOXA11-AS expression in ovarian cancer cells SKOV3, OVCAR3, A2780, and human ovarian surface epithelial cells IOSE-80. As demonstrated in Figure 1(b), the expression of HOXA11-AS in ovarian cancer cell lines (SKOV3, OVCAR3, A2780) was much greater than in normal ovarian cell lines (IOSE-80), particularly in OVCAR3 and A2780 cells. As a result, OVCAR3 and A2780 cell lines were chosen as representative ovarian cancer cell lines for the following research. The TCGA visualization database, UALCAN, exhibited that HOXA11-AS expression increases with ovarian cancer progression (Figure 1(c)). Kaplan–Meier survival curves suggested a poor prognosis of patients with high HOXA11-AS expression (Figure 1(d)).

### 3.1 Knockdown of lncRNA HOXA11-AS inhibits ovarian cancer cell malignant behavior

**Figure 2.**
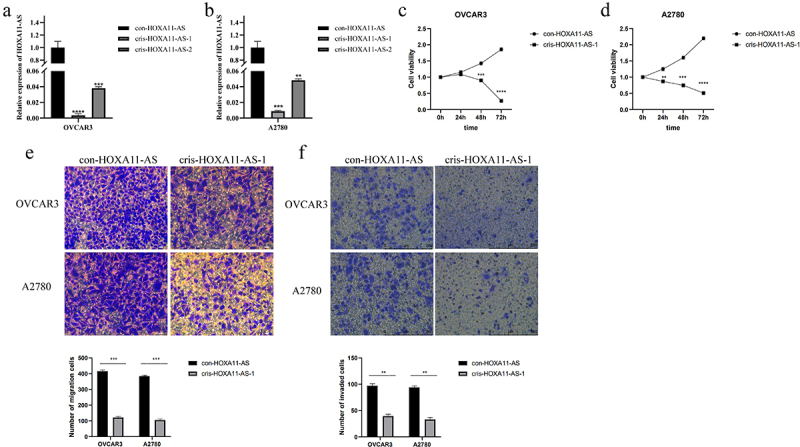
Inhibition of ovarian cancer cell malignant behavior after lncRNA HOXA11-AS knockdown. (a, b) The qRT-PCR assay showed the transfection efficiency of con- and cris-HOXA11-AS in OVCAR3 and A2780 cells. (c, d) The CCK-8 experiment exhibited that knockdown of HOXA11-AS significantly inhibited cell proliferation in OVCAR3 and A2780 cells. (e) Transwell assay was used to study the migration ability after the knockdown of HOXA11-AS in OVCAR3 and A2780 cells. (f) Transwell invasion assay was performed on cells transfected with cris-HOXA11-AS-1. The scale bar is 75 μm and 250 um, ** P < 0.01, *** P < 0.001 and **** P < 0.0001 vs. respective con-HOXA11-AS, and the results are from three independent repeated experiments.

To verify whether HOXA11-AS promotes cell proliferation, migration, and invasion in ovarian cancer cells, cris-HOXA11-AS and con-HOXA11-AS were transfected into OVCAR3 and A2780 cells, respectively. Transfection efficiency was evaluated by qRT-PCR (Figure 2(a, b)). In OVCAR3 and A2780 cells transfected with cris-HOXA11-AS, the expression of lncRNA HOXA11-AS was considerably lower than in cells transfected with con-HOXA11-AS. Because the cris-HOXA11-AS-1 group exhibited the highest interference efficiency, this group was used for subsequent experiments. CCK-8 experiment results exhibited that HOXA11-AS knockdown effectively blocked cell proliferation in OVCAR3 and A2780 cells (Figure 2(c, d)). Transwell assays showed pronounced inhibition in OVCAR3 and A2780 cell migration and invasion after HOXA11-AS downregulation (Figure 2(e, f)). The preceding evidence suggests that lncRNA HOXA11-AS may function as an oncogene that promotes the advancement of ovarian cancer.

### 3.2 Knockdown of HOXA11-AS expression enhances cisplatin sensitivity in ovarian cancer cells

**Figure 3.**
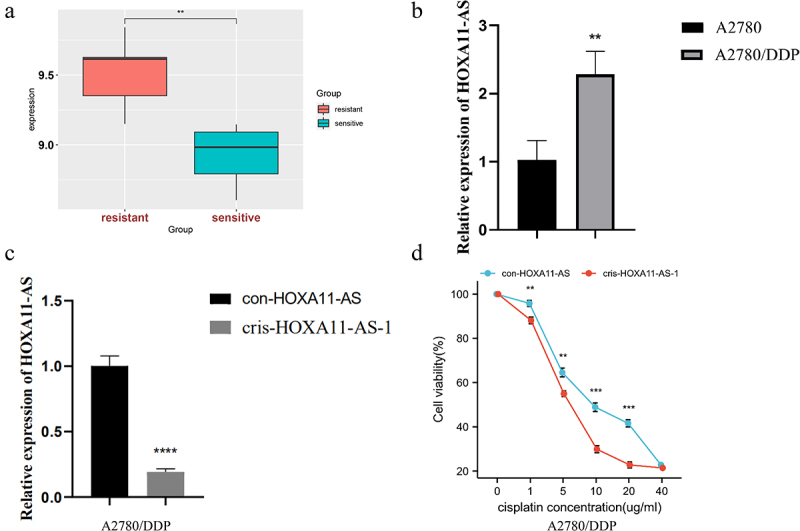
Knockdown of HOXA11-AS expression could enhance cisplatin sensitivity of ovarian cancer cells. (a) GSE15372 dataset exhibits that HOXA11-AS expression was higher in cisplatin-resistant A2780/DDP cells than cisplatin-sensitive A2780 cells. (b, c) qRT-PCR assays showed HOXA11-AS expression and transfection efficiency in A2780/DDP cells. (d) CCK-8 method showed the effect of low expression of HOXA11-AS on cisplatin sensitivity of A2780/DDP cells. ** P < 0.01, *** P < 0.001 and **** P < 0.0001 vs. respective con-HOXA11-AS, n = 3, Mean ± SD.

In the GSE15372 dataset, lncRNA HOXA11-AS expression was significantly upregulated in cisplatin-resistant ovarian cancer cells compared with cisplatin-sensitive cells (Figure 3(a)). Then, we detected HOXA11-AS expression in cisplatin-resistant A2780/DDP cells and A2780 cells by qRT-PCR. It was found that HOXA11-AS was markedly overexpressed in A2780/DDP cells (Figure 3(b)). We transfected con-HOXA11-AS, cris-HOXA11-AS-1 into A2780/DDP cells. The efficiency of lentivirus transfection was assessed by qRT-PCR (Figure 3(c)). The CCK-8 method was used to evaluate the cell viability of the cris-HOXA11-AS-1 and con-HOXA11-AS groups under differing cisplatin concentrations. The cell activity of the cris-HOXA11-AS group was found to be significantly lower than that of con-HOXA11-AS groups under differing cisplatin concentrations (1, 5, 10 and 20 μg/mL), indicating that HOXA11-AS could affect cisplatin sensitivity (Figure 3(d)).

### 3.3 HOXA11-AS knockdown led to cell cycle arrest, apoptosis, and autophagy

**Figure 4.**
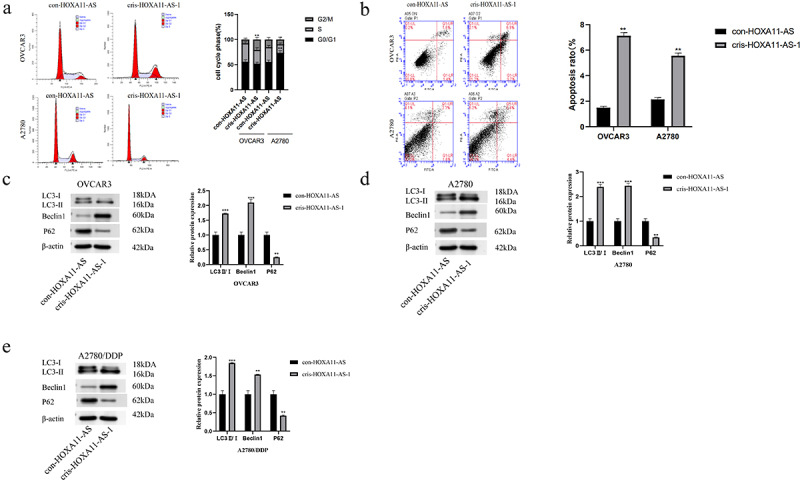
Knockdown of HOXA11-AS leads to cell cycle arrest and increased levels of apoptosis and autophagy. (a) Knockdown of HOXA11-AS could block cell cycle progression. (b) HOXA11-AS knockdown could increase the apoptosis rate of OVCAR3 and A2780 cells. (c-e) Western blot assay revealed the effect of HOXA11-AS knockdown on autophagy levels in OVCAR3, A2780, and A2780/DDP cells. The gray values of Western Blot bands by ImageJ software, and conduct statistical processing after normalization. ** P < 0.01, *** P < 0.001 vs. respective con-HOXA11-AS, n = 3, Mean ± SD.

Since HOXA11-AS greatly enhanced ovarian cancer cell proliferation, we investigated its involvement in cell cycle control. Flow cytometry was used to identify alterations in the cell cycle of ovarian cancer cells after HOXA11-AS knockdown. The effect of HOXA11-AS on cell growth was assessed. A significant G2/M phase block was observed in the transfected OVCAR3 cells, whereas a clear G0/G1 phase block was observed in A2780 cells (Figure 4(a)). As for cell apoptosis, the late apoptosis rate of the cris-HOXA11-AS-1 group exhibited a greater increase than that of the empty vector group (Figure 4(b)). Thus, the knockdown of HOXA11-AS expression could hinder the cell cycle of ovarian cancer cells and induced late apoptosis. Since autophagy is another mode of programmed cell death, we examined the changes in the level of intracellular autophagy following knockdown of HOXA11-AS. Autophagy-related proteins such as LC3, P62, and Beclin1 are commonly used to measure the level of intracellular autophagy, with high expression of LC3 II/I, Beclin1, and low expression of P62 indicating increased levels of intracellular autophagy. HOXA11-AS knockdown could lead to an increase in the protein level of Beclin1, a higher LC3II/I ratio, and a lower p62 protein expression (Figure 4(c-e)). The increase in LC3II/I ratio reflected the increase of autophagy formation or the obstruction of autolysosome formation. The changes in these protein levels showed that the level of autophagy increased after reducing the expression of HOXA11-AS.

### 3.4 Knockdown of HOXA11-AS affected ovarian cancer cells through autophagy

**Figure 5.**
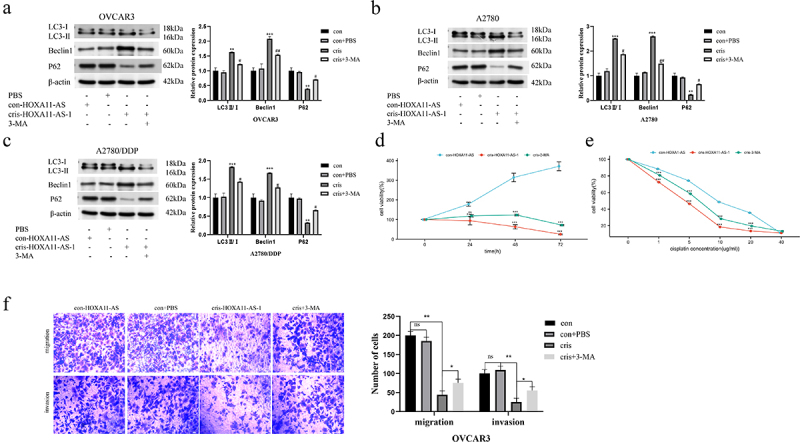
HOXA11-AS knockdown affects ovarian cancer cells through autophagy. (a-c) Western blot assay showed the effect of 3-methyladenine on autophagy levels in OVCAR3, A2780, and A2780/DDP cell lines transfected with cris-HOXA11-AS-1. (d) CCK-8 assay detected the cell activity after adding 3-methyladenine to cells with low HOXA11-AS expression. (e) CCK8 assay was used to detect the sensitivity of cris-HOXA11-AS-1 group cells to cisplatin after adding 3-methyladenine. (f) Transwell assay showed that 3-methyladenine enhanced the malignant behavior of cells with low HOXA11-AS expression. The scale bar is 75 μm. The gray values of Western Blot bands by ImageJ software, and conduct statistical processing after normalization. ^#^ P < 0.05, ^##^ P < 0.01 vs. respective cris group; ** P < 0.01, *** P < 0.001 vs. respective con-HOXA11-AS, n = 3, Mean ± SD.

After knockdown of the expression of HOXA11-AS in OVCAR3, A2780, and A2780/DDP cells, levels of the autophagic regulators P62, Beclin1, and LC3 were changed. Changes in autophagy levels can affect tumor progression and prognosis. Rescue experiments were undertaken to see whether suppressing autophagy in ovarian cancer cells was the cause of HOXA11-AS’s pro-carcinogenic effects on the disease. When 3-methyladenine was added to transfected cris-HOXA11-AS cells, intracellular autophagy levels were drastically reduced (Figure 5(a–c)). The CCK-8 experiment exhibited that 3-methyladenine could restore the cellular resistance to cisplatin under different cisplatin concentrations (Figure 5(e)). The CCK-8 and transwell experiments were used again in the cris-HOXA11-AS group of OVCAR3 cells, and 3-methyladenine was found to restore the slowdown of cellular proliferation, migration, and invasion caused by the knockdown of HOXA11-AS (Figure 5(d, f)). These findings imply that HOXA11-AS functions in a similar way as autophagy inhibitors, boosting the growth and advancement of ovarian cancer cells by lowering the degree of autophagy in them.

## 4. Discussion

Ovarian cancer is the most deadly gynecological malignancy [2] and a severe danger to women’s lives. Although contemporary surgery and platinum-based chemotherapy methods may greatly prolong the life expectancy of cancer patients, the high recurrence rate and chemoresistance seriously affect the follow-up treatment of patients. Platinum resistance is not only one of the main reasons for the high recurrence rate and low survival rate of ovarian cancer patients but also the main factor affecting the effect of secondary treatment [4]. This means that novel therapeutic targets and a better understanding of the molecular processes of cisplatin resistance are critical in ovarian cancer treatment and prevention, respectively.

Molecular targeted therapy is a promising treatment strategy for prolonging the survival time of patients with cancer [36]. Compared with chemotherapy alone, chemotherapy combined with molecularly targeted drugs can play a stronger anti-tumor role and help prolong the survival time of patients [37]. However, molecularly targeted drugs can also bring a series of side effects, such as anemia, leukopenia, digestive tract perforation, etc [38]. How to improve the quality of life of patients while achieving efficacy still needs us to continue to explore more therapeutic targets and targeted drugs.

LncRNA is a kind of non-protein coding RNA with a length of more than 200 nucleotides (nt) in the non-coding RNA family [11]. LncRNAs have recently been shown to impact tumor growth and prognosis by influencing drug metabolism, autophagy, apoptosis, and the epithelial-mesenchymal transition, according to current research [39–42]. For example, lncRNA UCAI is an oncogene that is resistant to cisplatin in gastric cancer. It can activate the PI3K/AKT pathway by regulating the EZH2 gene expression, thereby making patients with gastric cancer resistant to cisplatin [43]. lncRNA EGOT has also been reported as a prognostic marker for colon cancer that can facilitate cancer cell malignant behavior by inhibiting autophagy and the degree of apoptosis in colon cancer cells [44]. One of the oldest and most conserved groups of HOX family known to have an impact on cell metabolism and apoptosis has been linked to cancer development and progression [17]. As a member of HOX family, HOXA11-AS is an antisense transcript of the HOXA11 gene that serves as a tumor-promoting factor for many malignancies. For example, HOXA11-AS upregulation can be detected in gastric cancer tissues and cells and mainly fuels cell proliferation via reduced apoptosis and uncontrolled cell cycle progression [45]. More importantly, Lin et al. found that the expression of HOXA11-AS was up-regulated in nasopharyngeal carcinoma drug-resistant cells, and the silencing of HOXA11-AS reversed the drug resistance of nasopharyngeal carcinoma to cisplatin [46]. HOXA11-AS can also act as ceRNA to regulate miR-518a/SPATS2L expression and promote cisplatin resistance in laryngeal squamous cell carcinoma [47]. In ovarian cancer, a study claimed that HOXA11-AS might operate as a tumor promoter in epithelial ovarian cancer, and qRT-PCR revealed that the expression of HOXA11-AS was 77-fold greater in epithelial ovarian cancer than in normal ovarian tissue [28]. Moreover, increased HOXA11-AS expression was substantially linked with higher histological grade, higher tumor antigen 125 (CA125) levels, and worse patient survival [28]. However, less research had been conducted on HOXA11-AS in ovarian cancer, and the effect of HOXA11-AS on ovarian cancer progression and cisplatin resistance is not clear. The above existing evidence emphasizes the importance of HOXA11-AS in regulating the progression of ovarian cancer. Therefore, there is an urgent need to better comprehend the regulation mechanisms of HOXA11-AS in ovarian cancer to create effective ovarian cancer therapeutics. In this work, we focused on the expression level of HOXA11-AS in ovarian cancer cell lines and highlighted its multifaceted role in ovarian cancer cell function.

In the present study, we analyzed multiple chip sequencing files in the GEO database to perform qRT-PCR experiments on normal ovarian and ovarian cancer cells and observed a significant increase in HOXA11-AS expression in the ovarian cancer tissues and cells compared with normal controls. Moreover, HOXA11-AS expression in the cisplatin-resistant group was found to be markedly elevated in ovarian cancer cells. Due to HOXA11-AS’s significance in modulating tumor cell function and predicting overall survival [48], we hypothesized that this lncRNA may act as a tumor promoter in ovarian cancer and as a prognostic marker. Using the UALCAN visualization portal of TCGA, it was determined that the expression of HOXA11-AS increased with the advancement of ovarian cancer. Moreover, Kaplan-Meier curves demonstrated the potential predictive value of HOXA11-AS for ovarian cancer survival. Therefore, we predicted that the overexpression of HOXA11-AS indicated a bad prognosis in individuals with ovarian cancer. In light of the dysregulated expression of HOXA11-AS in ovarian cancer, we investigated whether HOXA11-AS influences the biological activity of ovarian cancer cells. Firstly, we constructed the HOXA11-AS interference lentiviral vector and the corresponding empty vector. HOXA11-AS expression pronouncedly dropped after HOXA11-AS knockdown, alongside suppressed cell proliferation, migration, and invasion. Additionally, low HOXA11-AS expression causes G0/G1 cell cycle arrest, increases the apoptosis rate, and improves the cisplatin sensitivity of ovarian cancer cells. HOXA11-AS may be related to the progression of ovarian malignant tumors and used to develop targeted agents and predictive gene signatures in future clinical practice.

As a catabolic pathway in the human body, autophagy can offer alternative energy resources from degraded protein aggregates or polymers and organelles [49]. Autophagy may be a double-edged sword for drug-resistant tumors, which participates in the development of drug-resistant tumors, protects cancer cells from survival in a chemotherapy environment, and can also kill drug-resistant cancer cells with inactive apoptosis pathways. Currently, studies have shown that apoptosis and autophagy have synergistic anti-tumor effects. Many chemotherapy drugs can cause autophagy and apoptosis in cells at the same time. Under certain conditions, autophagy can become a clearing agent for the apoptosis blocking signal pathway and promote apoptosis of drug-resistant tumors [50]. Recent research has shown that the HOX family is also involved in the regulation of autophagy in tumor cells. For instance, the autophagy-related lncRNA HOTAIRM1 is implicated in the regulation of autophagy levels in myeloid cells and drives the development of myeloid leukemia [51]. It has been hypothesized that autophagy-related signaling pathways are primarily responsible for the regulating effect of HOXA11-AS on autophagy. The PI3K/AKT/mTOR signaling pathway is one of the primary signaling mechanisms involved in autophagy regulation. Studies have shown that decreasing the expression of HOXA11-AS in skin cancer cells may inhibit the proliferation, migration, and invasion of skin cancer cells by inhibiting the PI3K/AKT/mTOR signaling pathway [52]. Rarely documented, however, is the significance of HOXA11-AS in the control of autophagy in ovarian cancer cells. The change in the LC3-II/I ratio is a crucial indicator for changes in autophagy levels, and P62 and Beclin1 are also the markers of autophagy [53]. When autophagy is formed, the cytoplasmic LC3 protein enzymatically cleaves small segments of polypeptides to form LC3I, which eventually embeds into the autophagosome membrane to form LC3II, so the magnitude of the LC3II/I value can be used to measure the level of autophagy. p62 protein can be specifically degraded by autophagy, so P62 levels are often used as a marker of inhibition of autophagy or defective autophagic degradation [53]. Beclin1 is an autophagy positive regulatory molecule, and its high expression represents the activation of intracellular autophagic effects. Therefore, high expression of LC3, Beclin1, and low expression of P62 are commonly used to represent enhanced levels of intracellular autophagy in experiments to detect autophagic flow. We found that knockdown of HOXA11-AS resulted in increased expression of autophagy-related proteins (Beclin1, LC3-II/I) and decreased expression of P62 proteins in cells by Western blot assay. There is evidence to suggest that HOXA11-AS has a major impact on the degree of autophagy inside cells, which may be the way it regulates the biological activity of ovarian cancer cells. To verify our hypothesis, we conducted a rescue experiment at the autophagy level. The addition of 3-methyladenine, an autophagy inhibitor, to cisplatin-resistant ovarian cancer cells stably transfected with lentivirus was found to decrease the LC3-II/I ratio and Beclin1 expression. Increasing the expression of P62 protein partially reversed the inhibitory effect of knockdown of HOXA11-AS on ovarian cancer cell function. Thus, changing the level of autophagy provides a novel idea for studying how HOXA11-AS facilitates ovarian cancer progression. However, animal experiments were not conducted and the expression of HOXA11-AS in clinical samples was not detected in this study. Therefore, we plan to verify the expression and cancer-promoting effect of HOXA11-AS through animal experiments and clinical samples in future studies, and further explore the regulatory mechanism of HOXA11-AS on autophagy.

## 5. Conclusion

In conclusion, we found that the expression of HOXA11-AS was up-regulated in ovarian cancer tissues and cisplatin-resistant cells, and HOXA11-AS expression increase was predictive of a poor prognosis (log-rank P = 0.00089). Knockdown of HOXA11-AS expression in ovarian cancer cells significantly inhibited the malignant behavior of ovarian cancer cells and increased their sensitivity to cisplatin(P < 0.05). Knockdown of HOXA11-AS promotes the level of intracellular autophagy, thereby inhibiting the growth and drug resistance of tumor cells(P < 0.05). Consequently, the impact of HOXA11-AS on cellular autophagy may serve as a viable therapeutic target and a novel treatment method for ovarian cancer.

## Supplementary Material

Supplemental Material

## Data Availability

The datasets used and/or analyzed during the current study are available from the corresponding author on reasonable request.
