# Supplementary material for: Long non-coding RNA HOXA11-AS knockout inhibits proliferation and overcomes drug resistance in ovarian cancer
Source: Bioengineered. 2022 Jun 15;13(5):13893–905. doi: 10.1080/21655979.2022.2086377 (PMC9276031; doi:10.1080/21655979.2022.2086377)
Supplement: Supplemental Material [file KBIE_A_2086377_SM9126.pptx]

## Slide 1
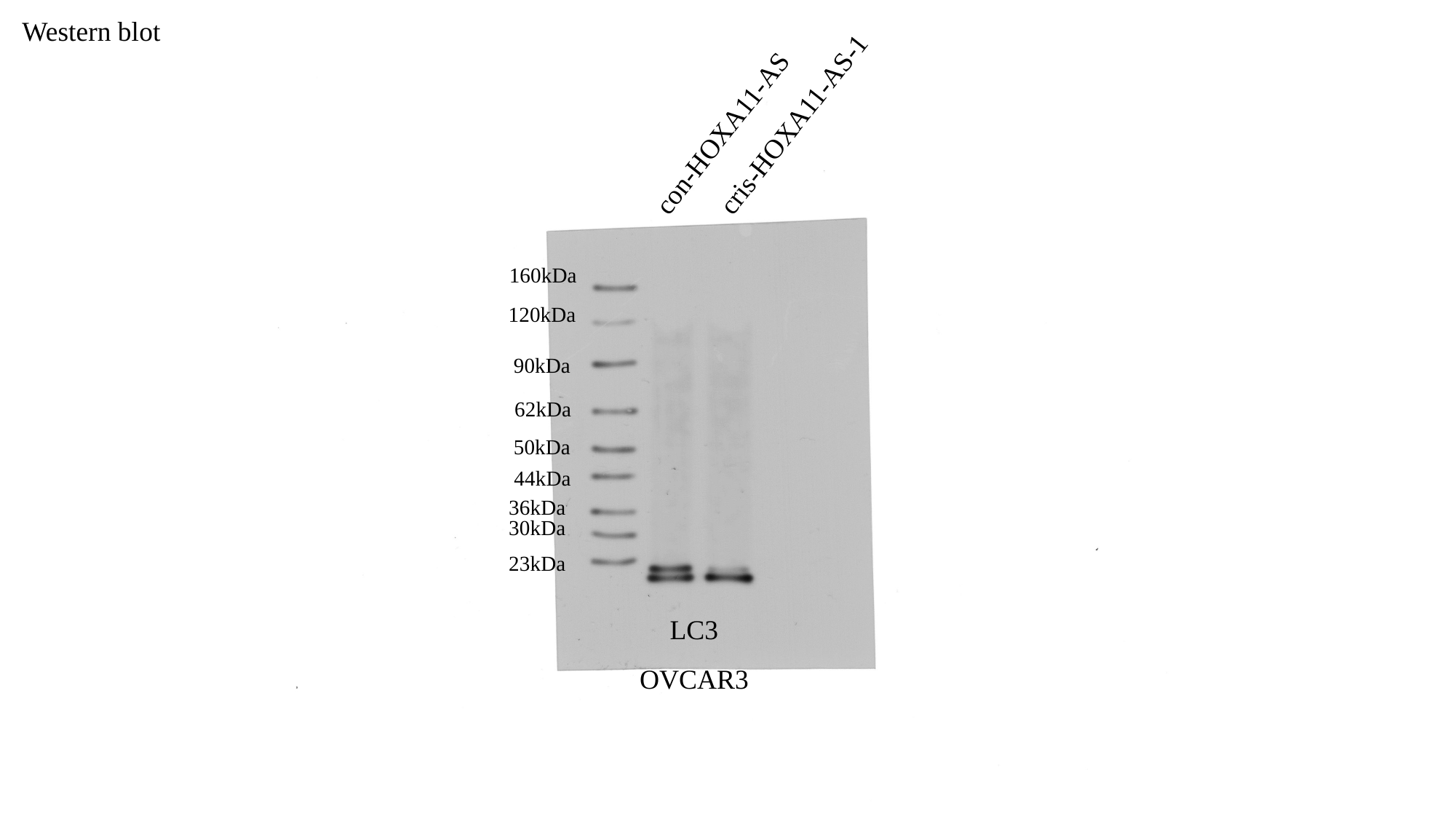

Western blot
cris-HOXA11-AS-1
con-HOXA11-AS
160kDa
120kDa
90kDa
62kDa
50kDa
44kDa
36kDa
30kDa
23kDa
LC3
OVCAR3

## Slide 2
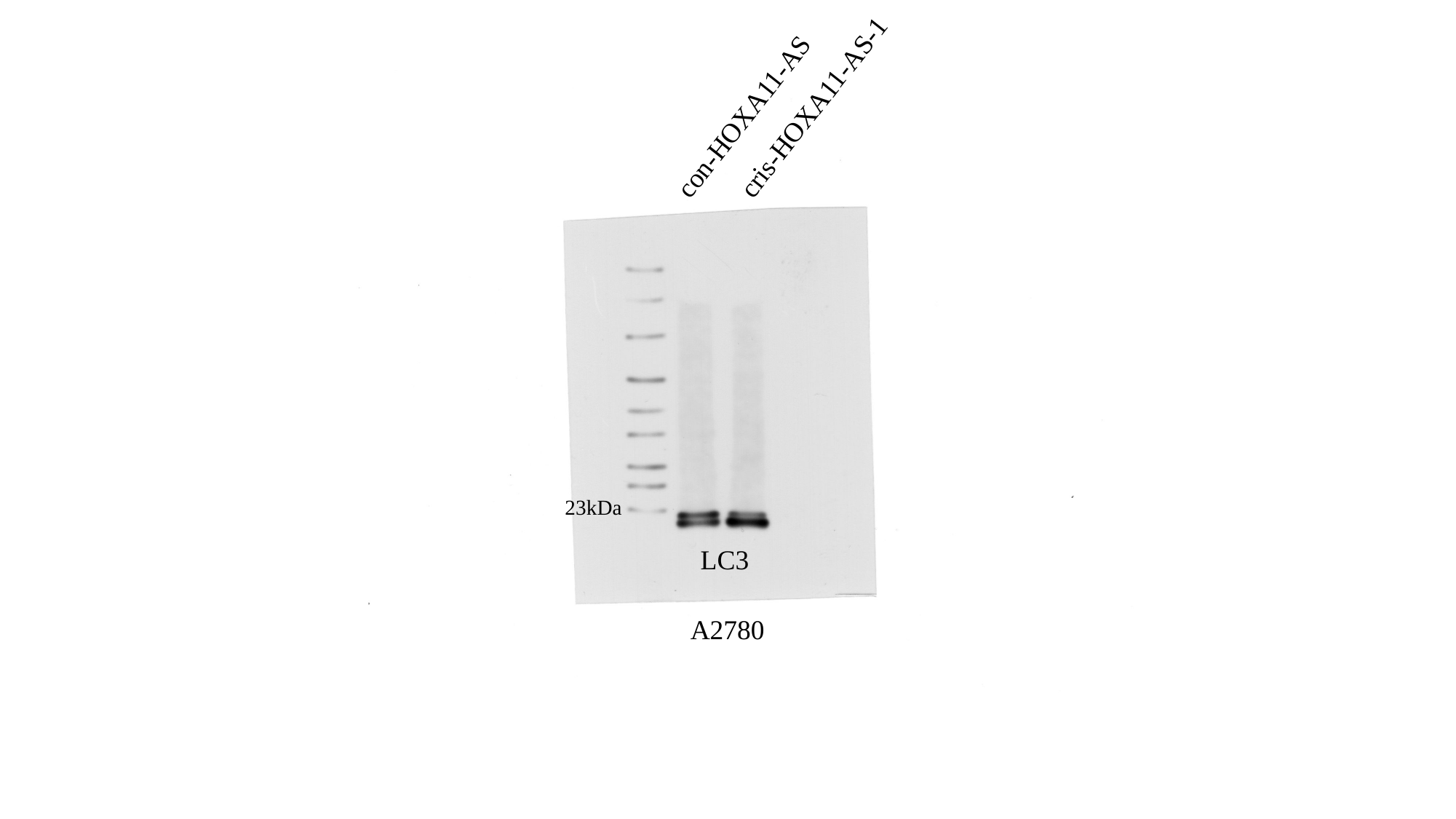

cris-HOXA11-AS-1
con-HOXA11-AS
23kDa
LC3
A2780

## Slide 3
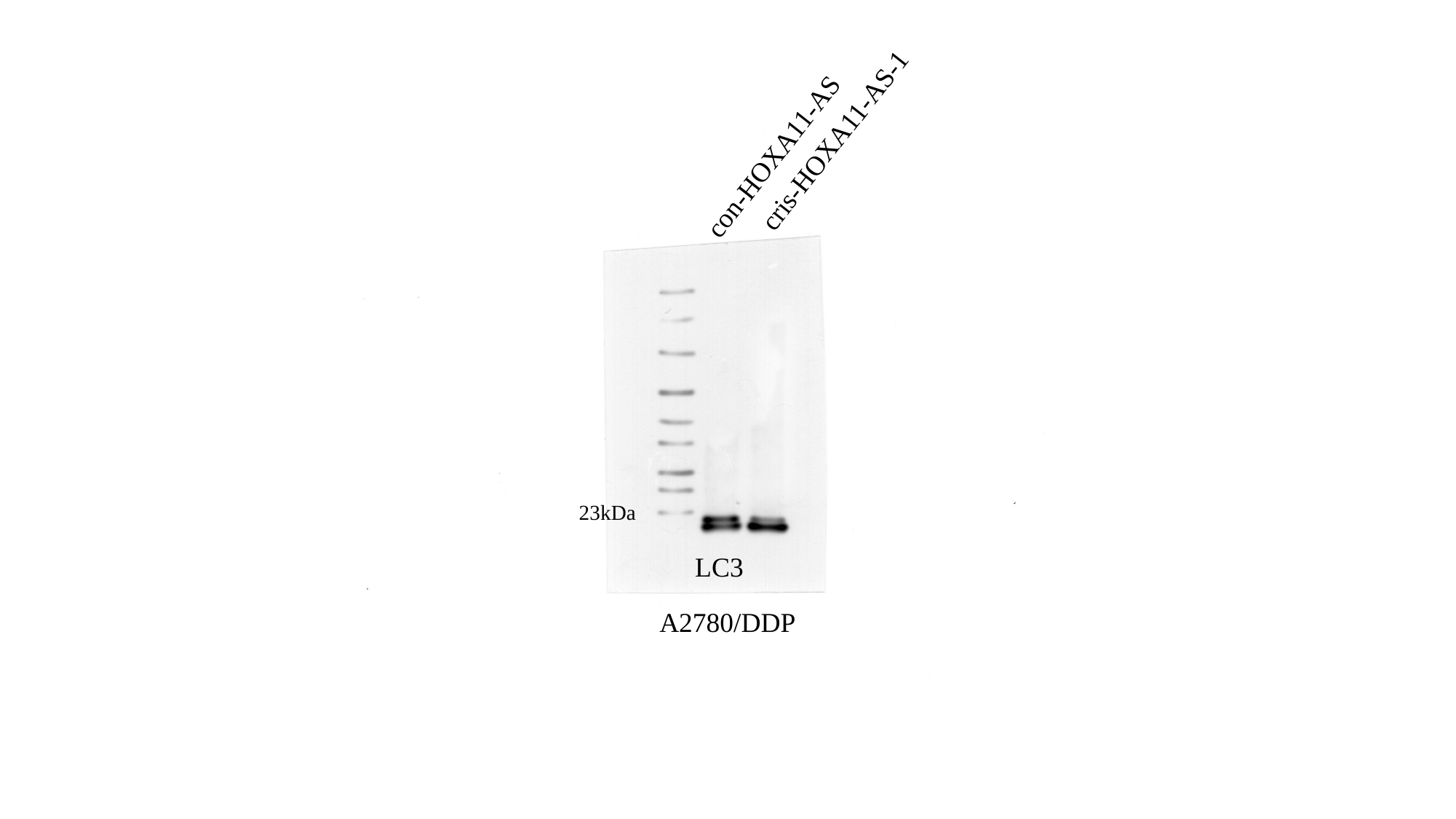

cris-HOXA11-AS-1
con-HOXA11-AS
23kDa
LC3
A2780/DDP

## Slide 4
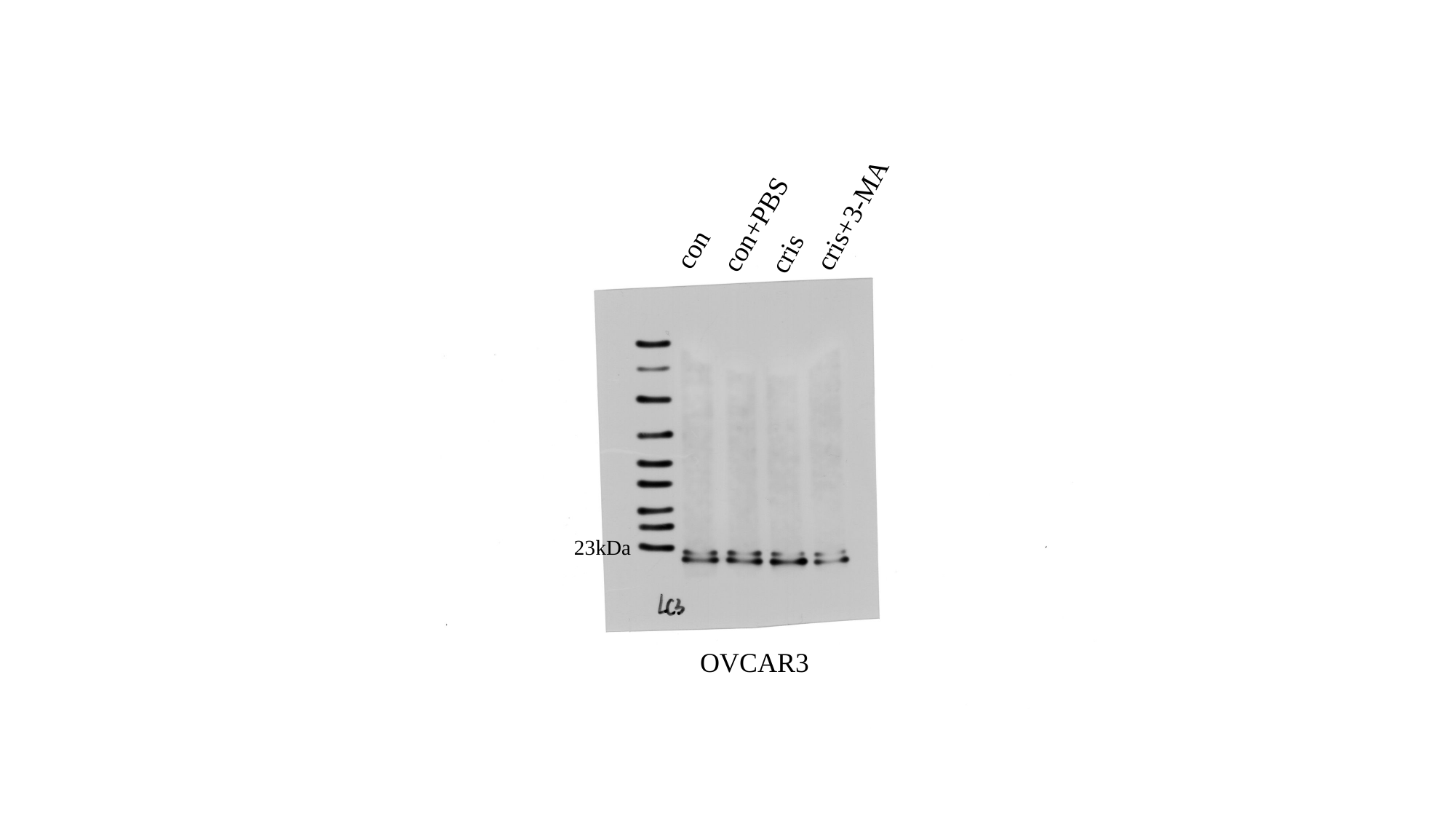

cris+3-MA
con+PBS
con
cris
23kDa
OVCAR3

## Slide 5
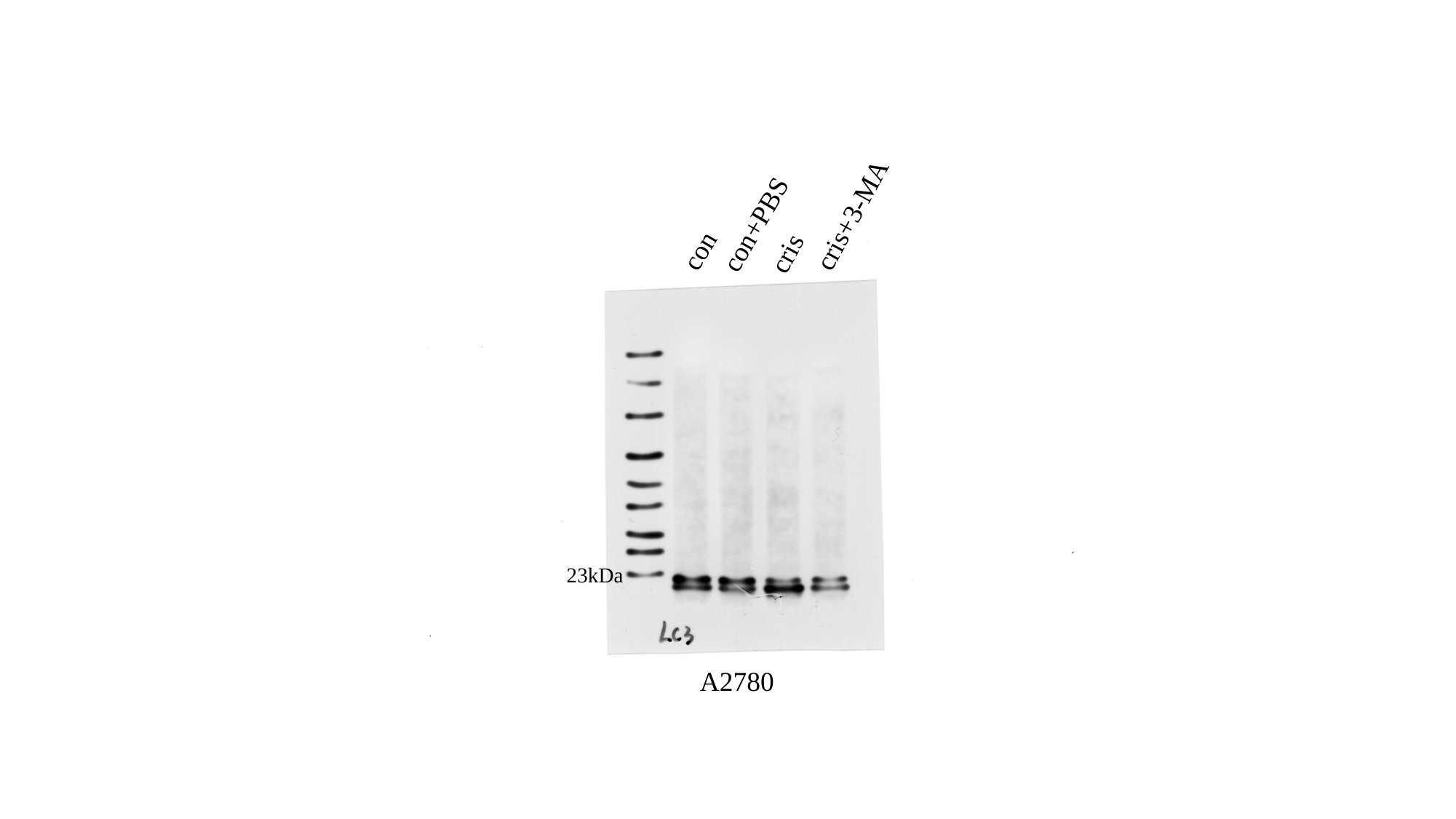

cris+3-MA
con+PBS
con
cris
23kDa
A2780

## Slide 6
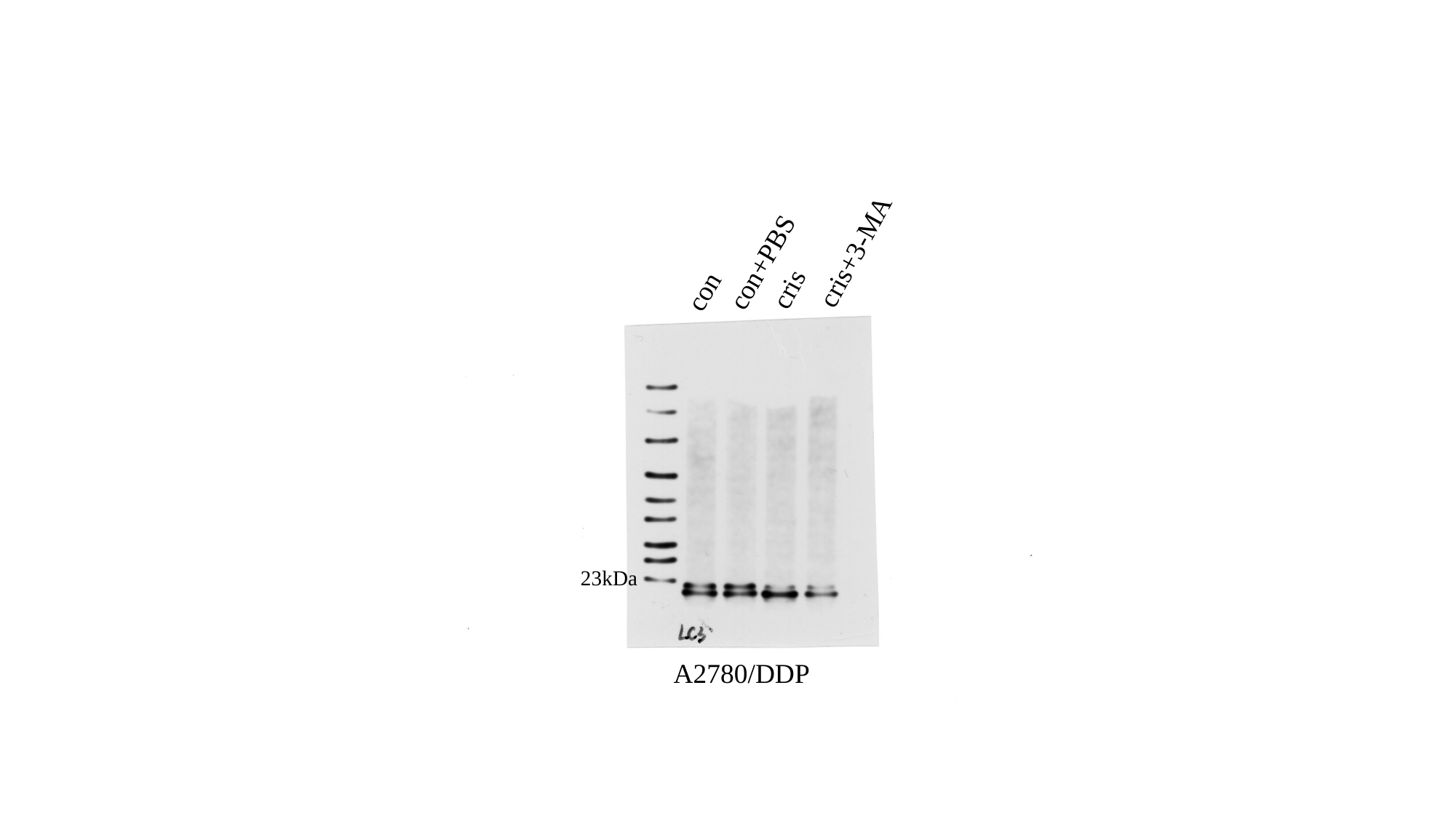

cris+3-MA
con+PBS
cris
con
23kDa
A2780/DDP

## Slide 7
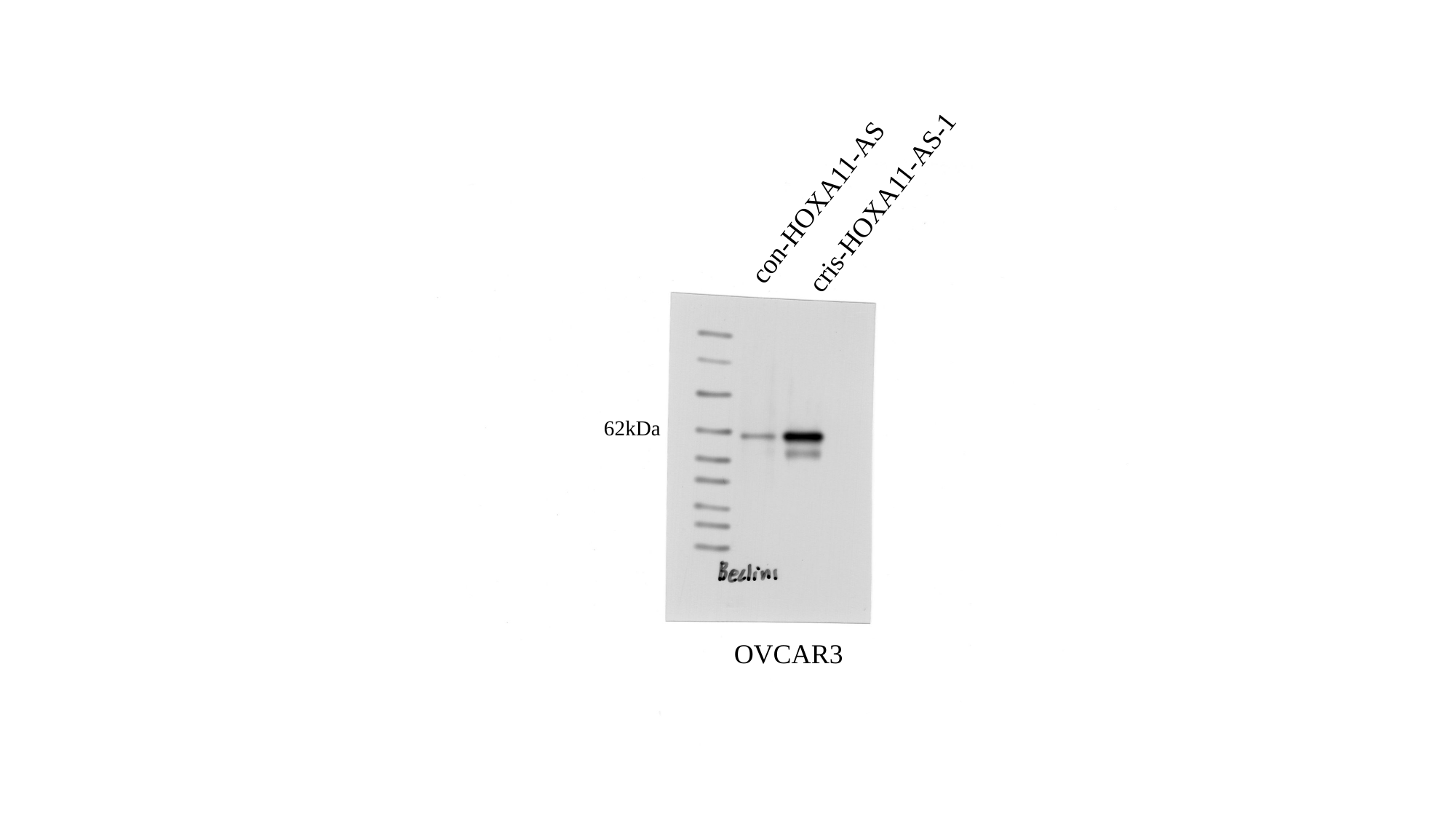

cris-HOXA11-AS-1
con-HOXA11-AS
62kDa
OVCAR3

## Slide 8
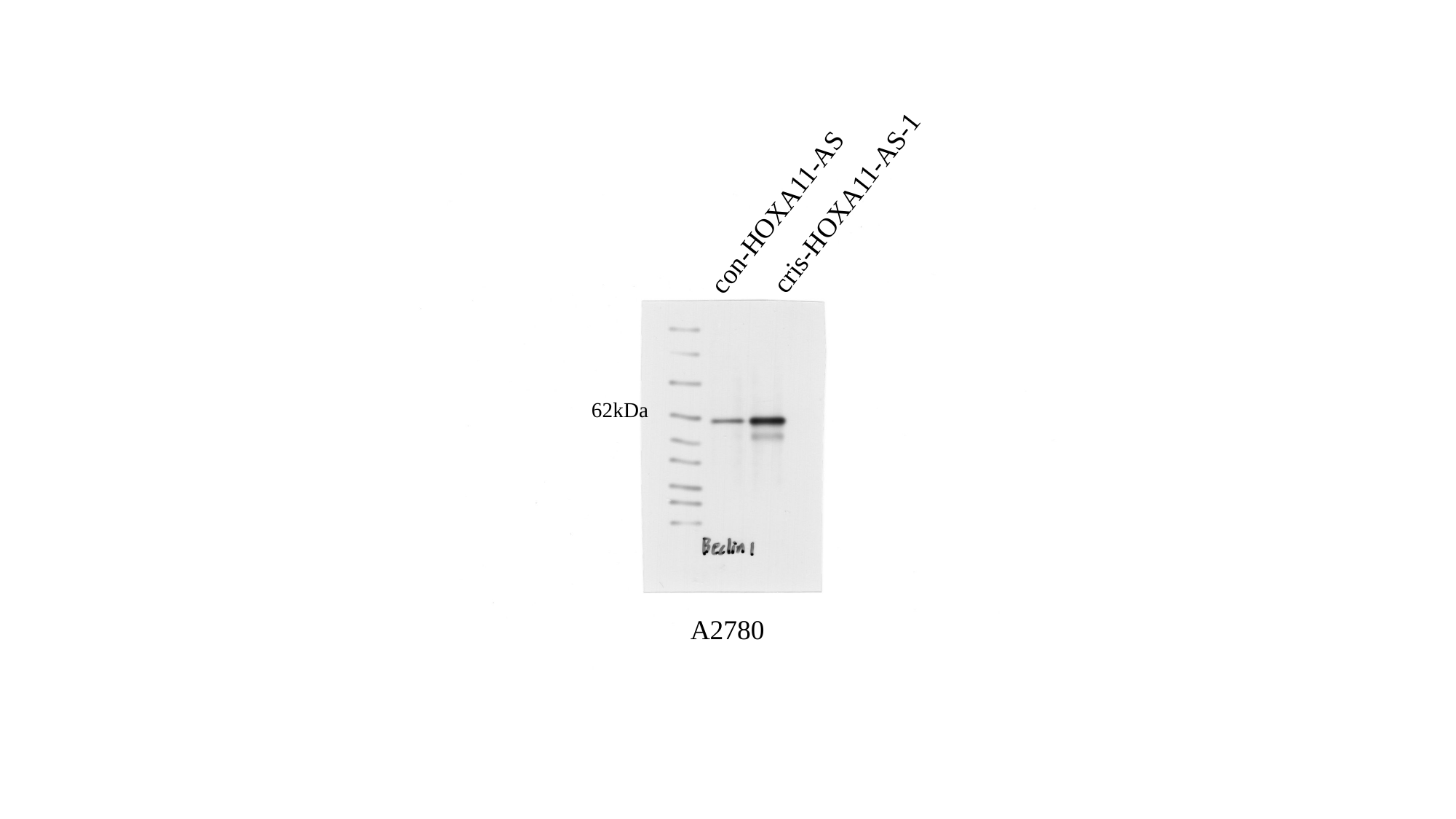

cris-HOXA11-AS-1
con-HOXA11-AS
62kDa
A2780

## Slide 9
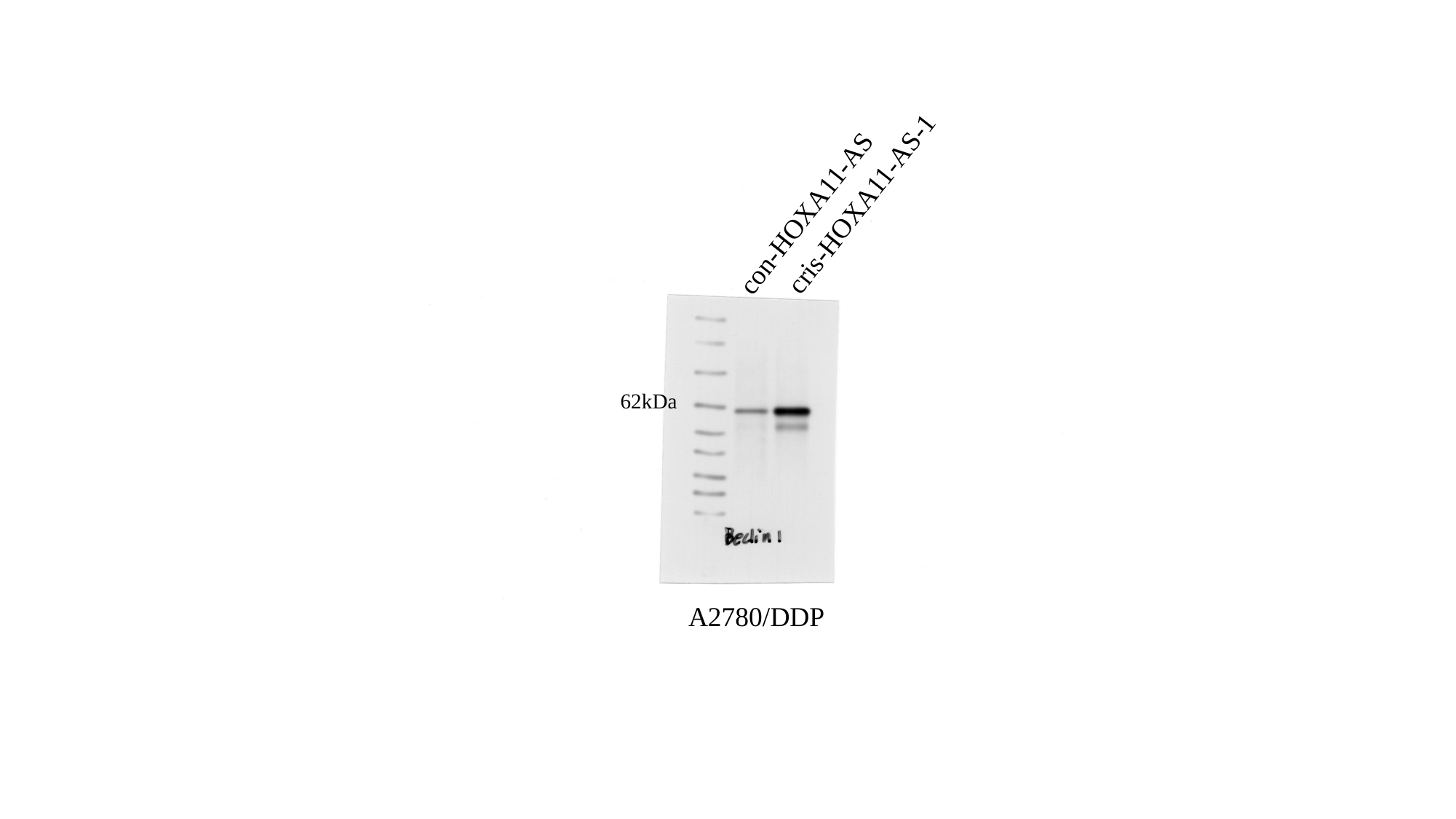

cris-HOXA11-AS-1
con-HOXA11-AS
62kDa
A2780/DDP

## Slide 10
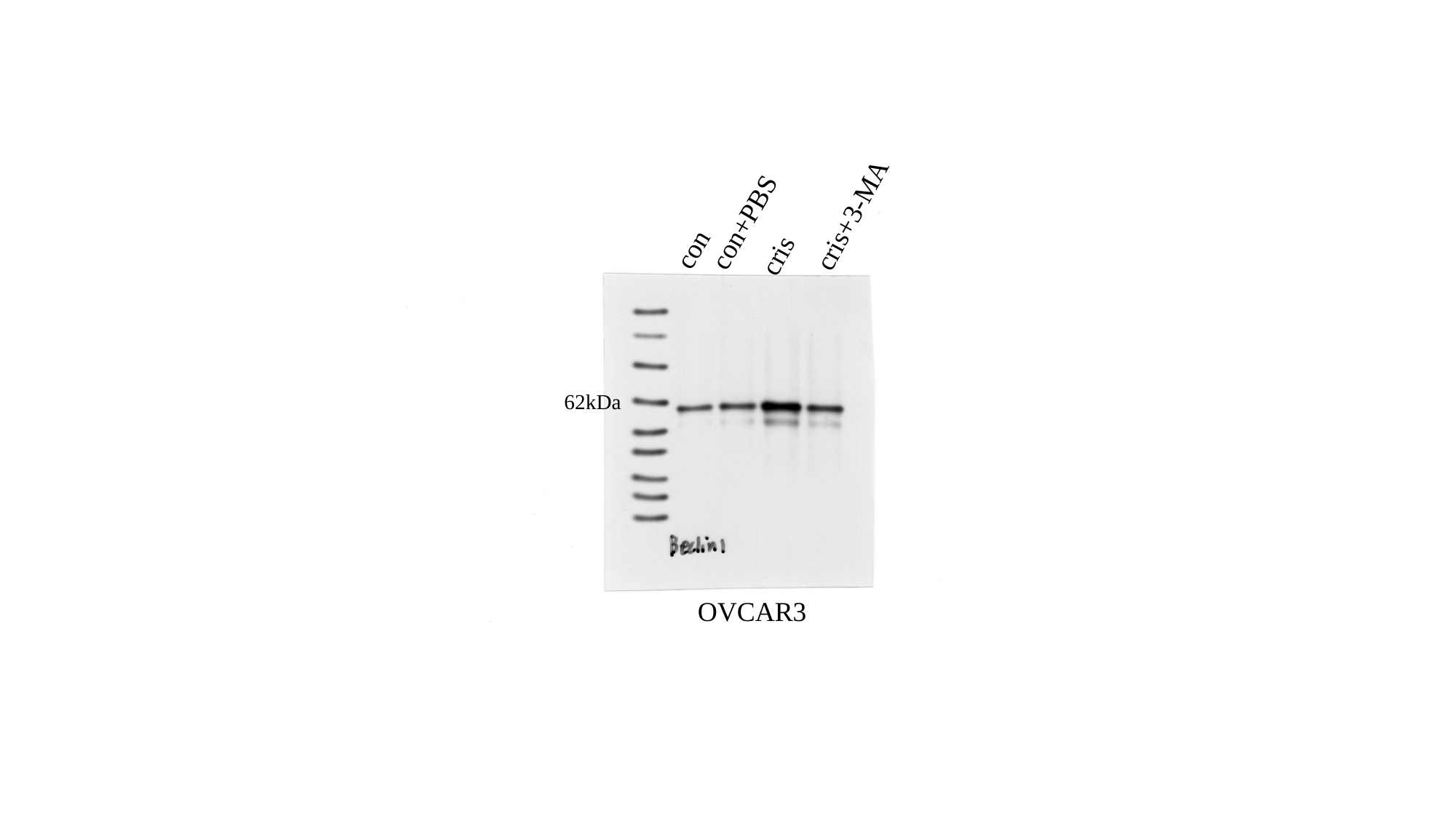

cris+3-MA
con+PBS
con
cris
62kDa
OVCAR3

## Slide 11
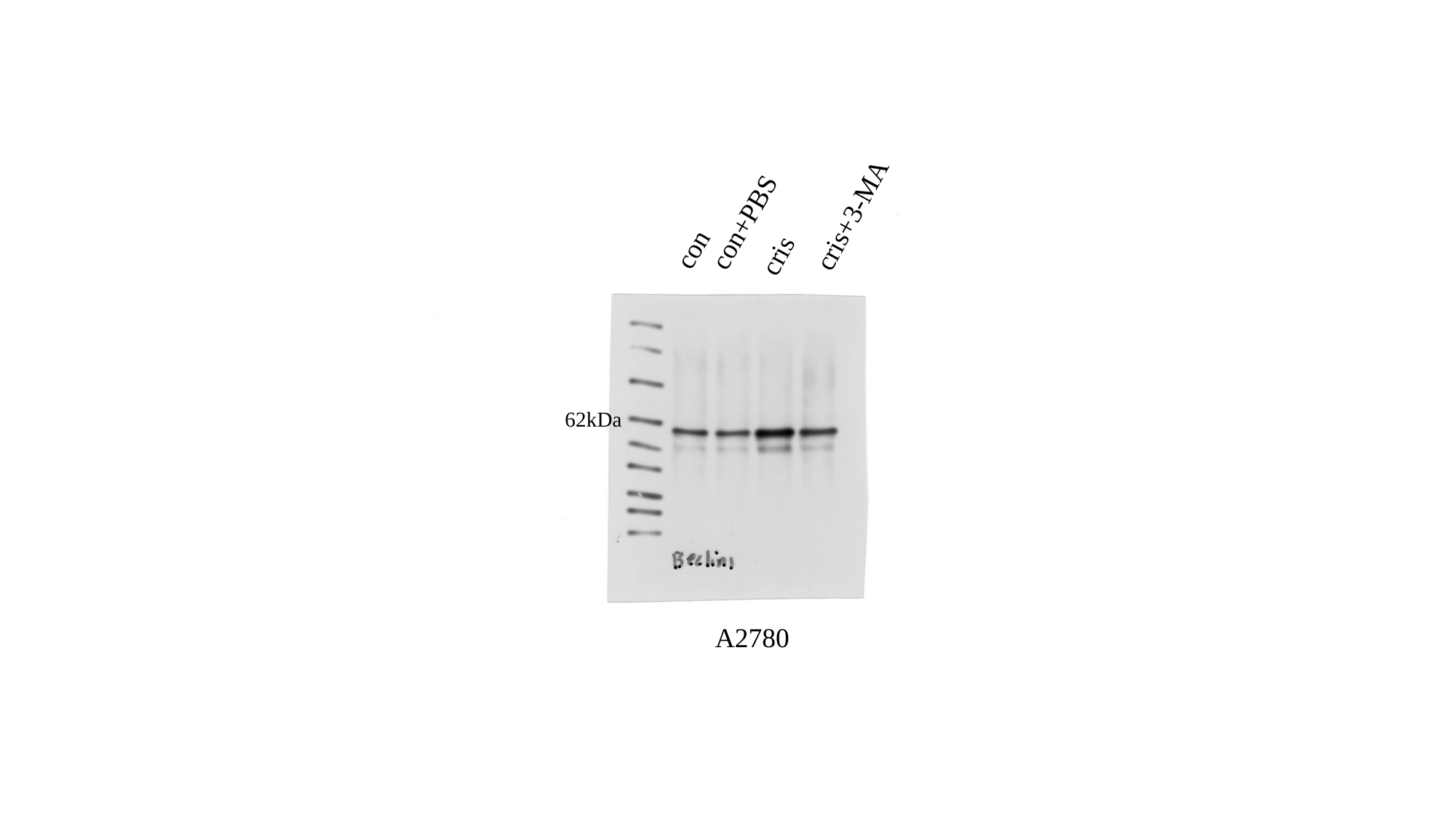

cris+3-MA
con+PBS
con
cris
62kDa
A2780

## Slide 12
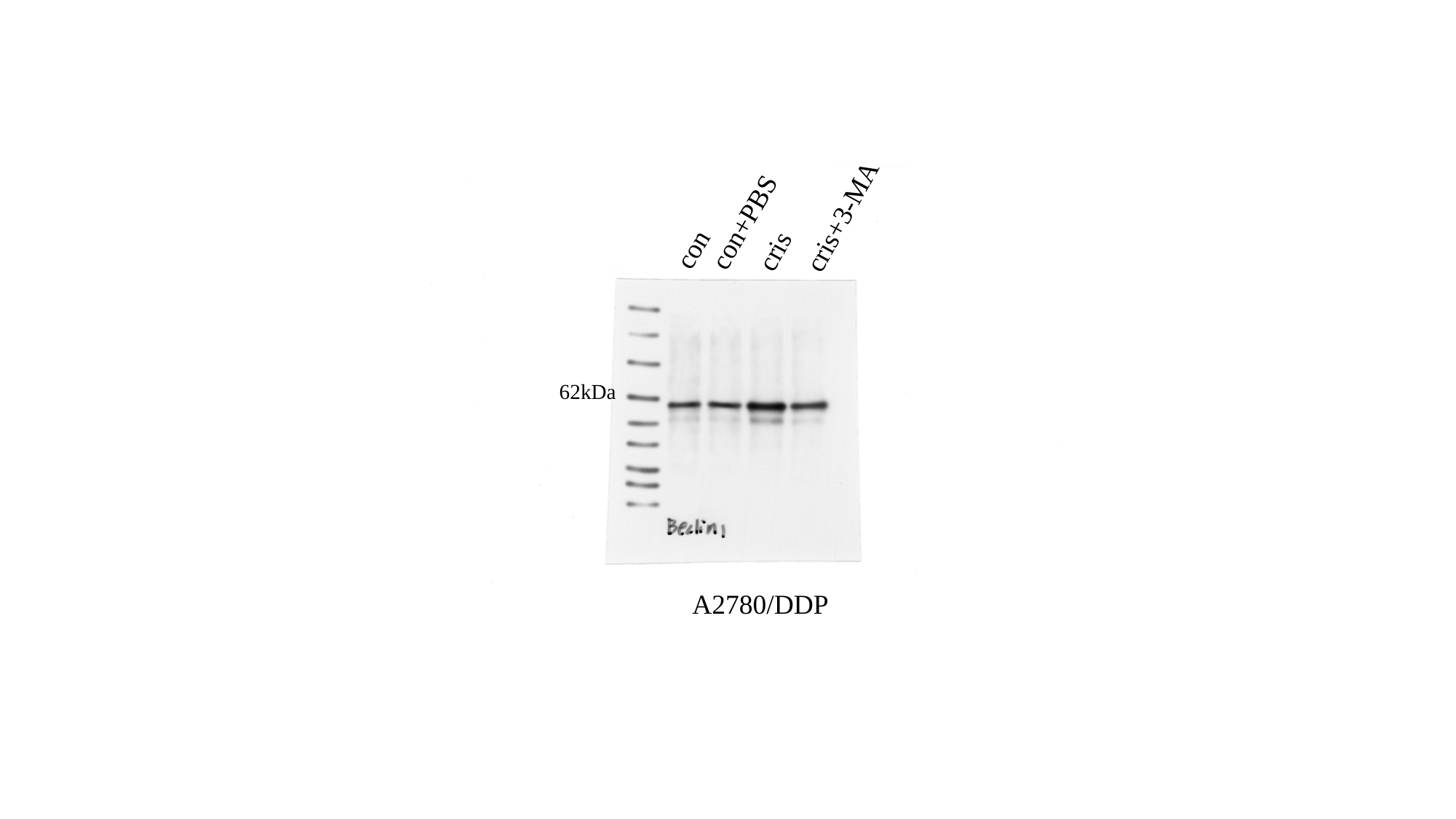

cris+3-MA
con+PBS
con
cris
62kDa
A2780/DDP

## Slide 13
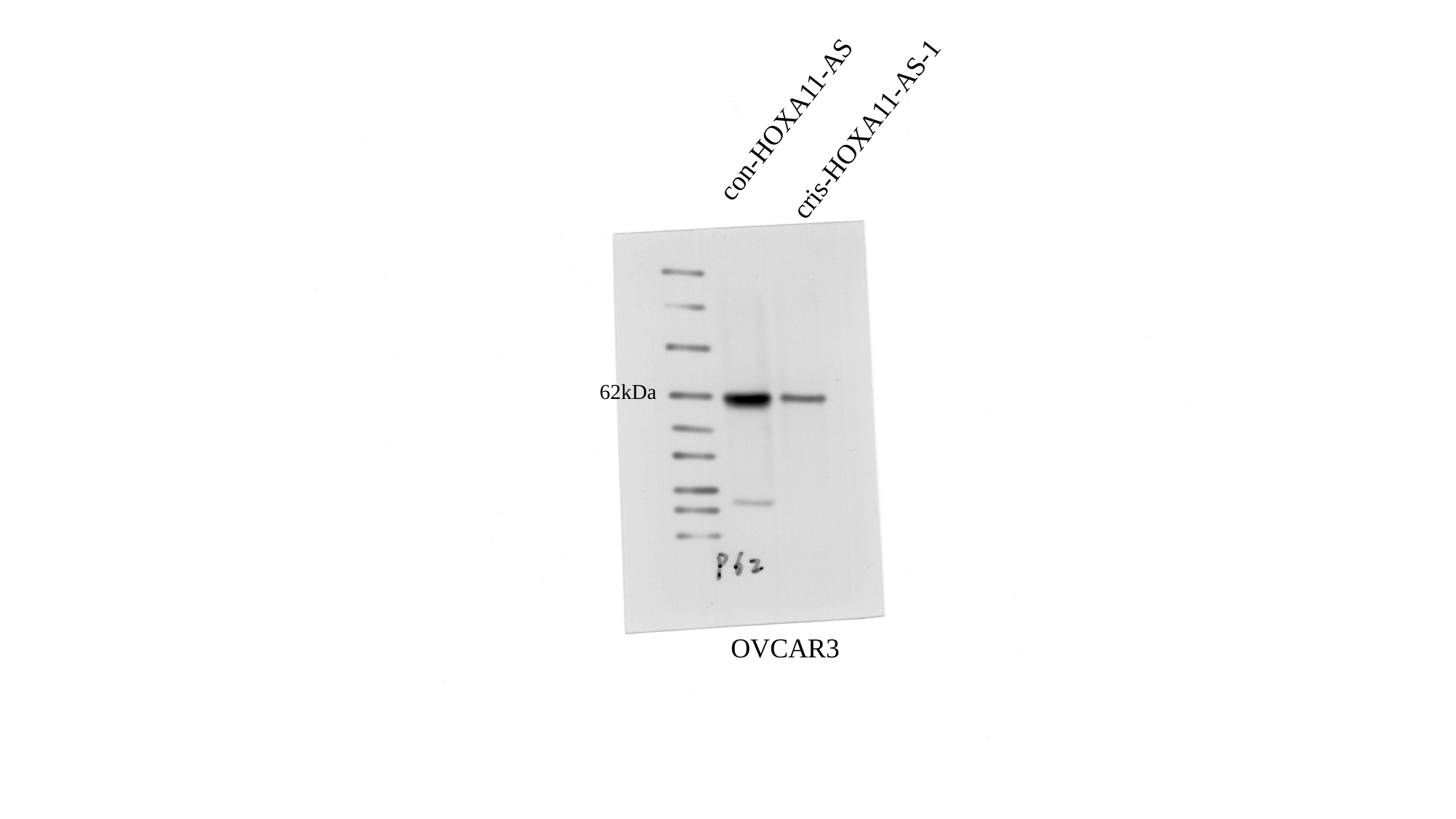

con-HOXA11-AS
cris-HOXA11-AS-1
62kDa
OVCAR3

## Slide 14
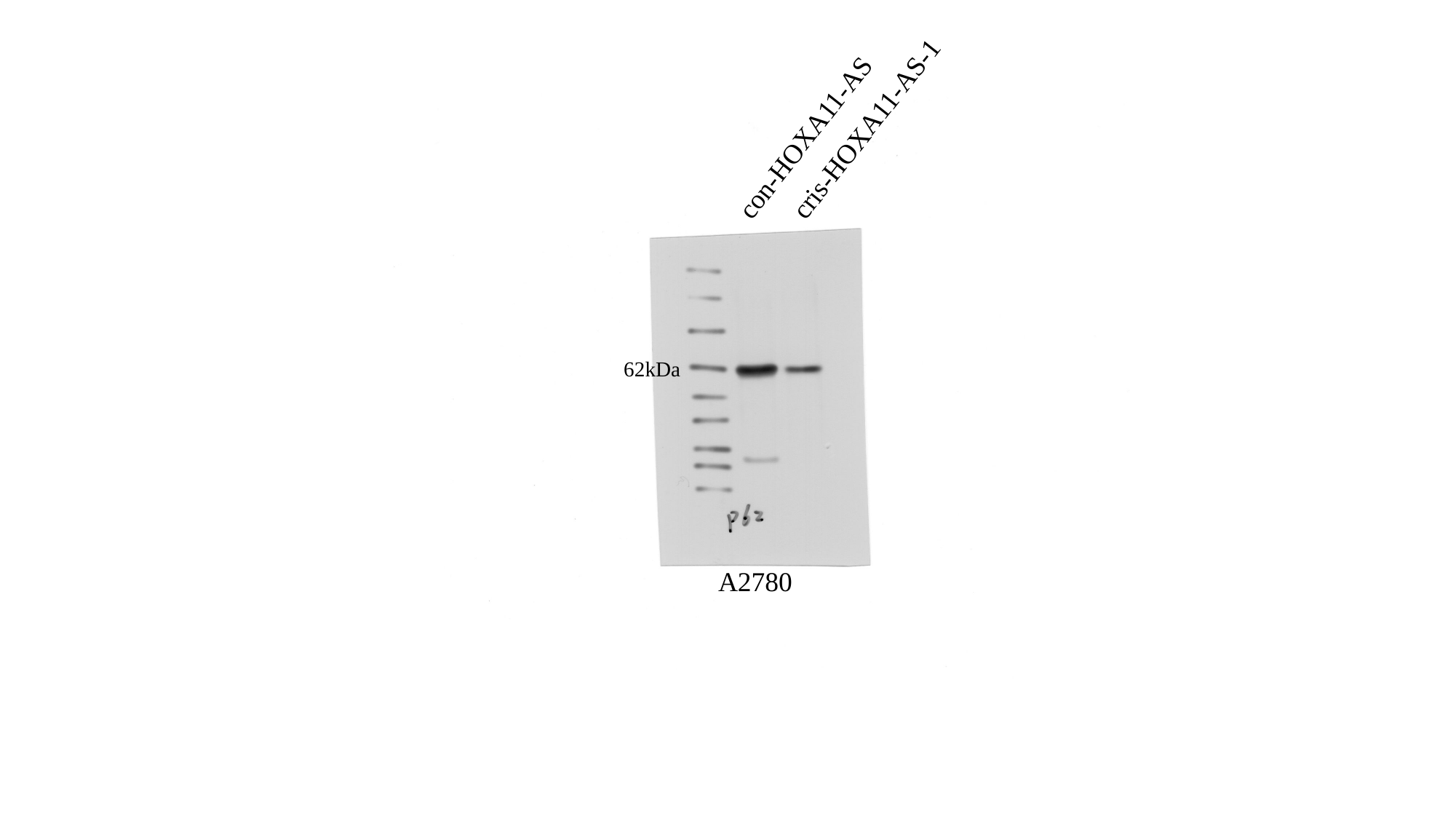

cris-HOXA11-AS-1
con-HOXA11-AS
62kDa
A2780

## Slide 15
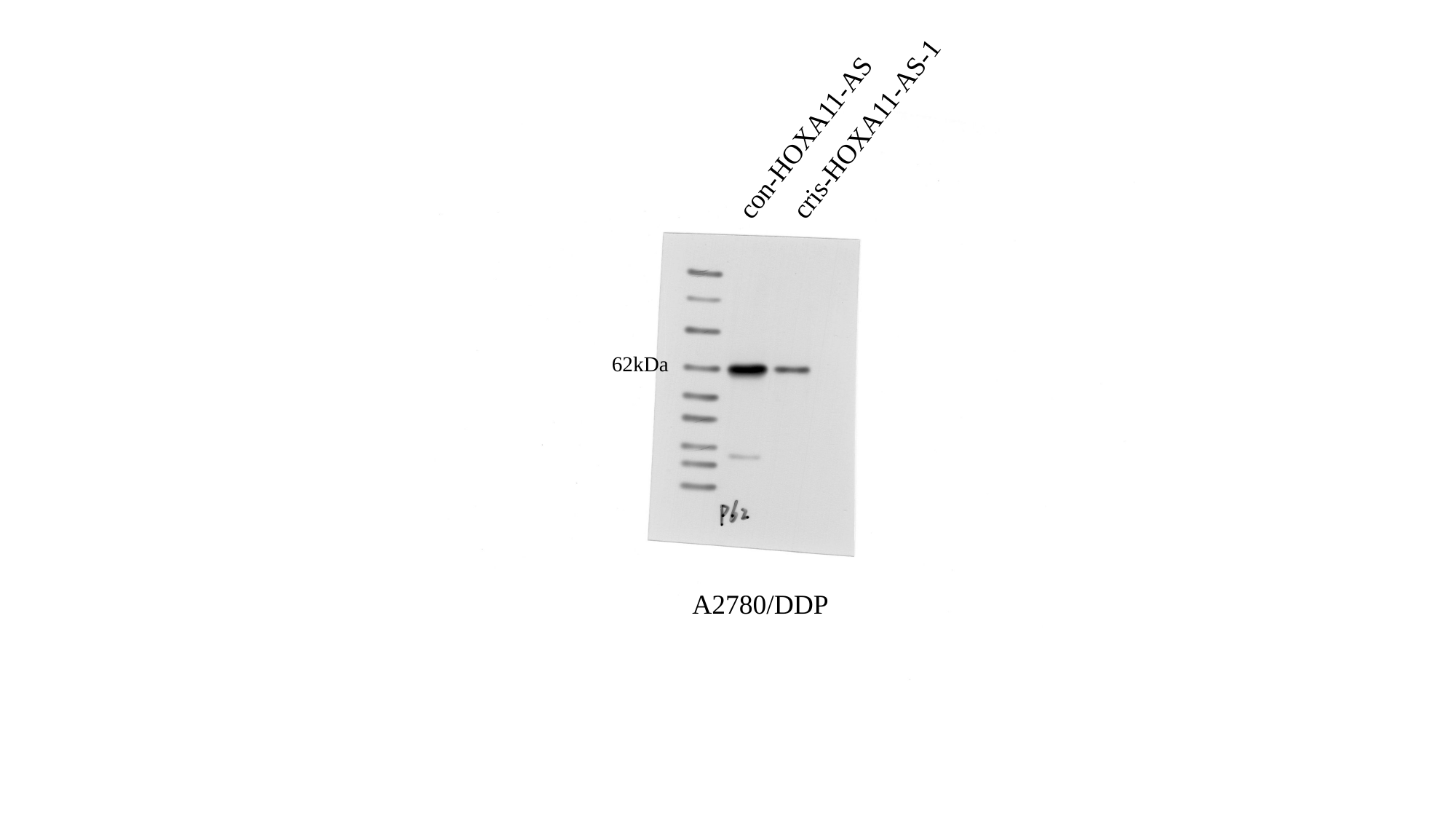

cris-HOXA11-AS-1
con-HOXA11-AS
62kDa
A2780/DDP

## Slide 16
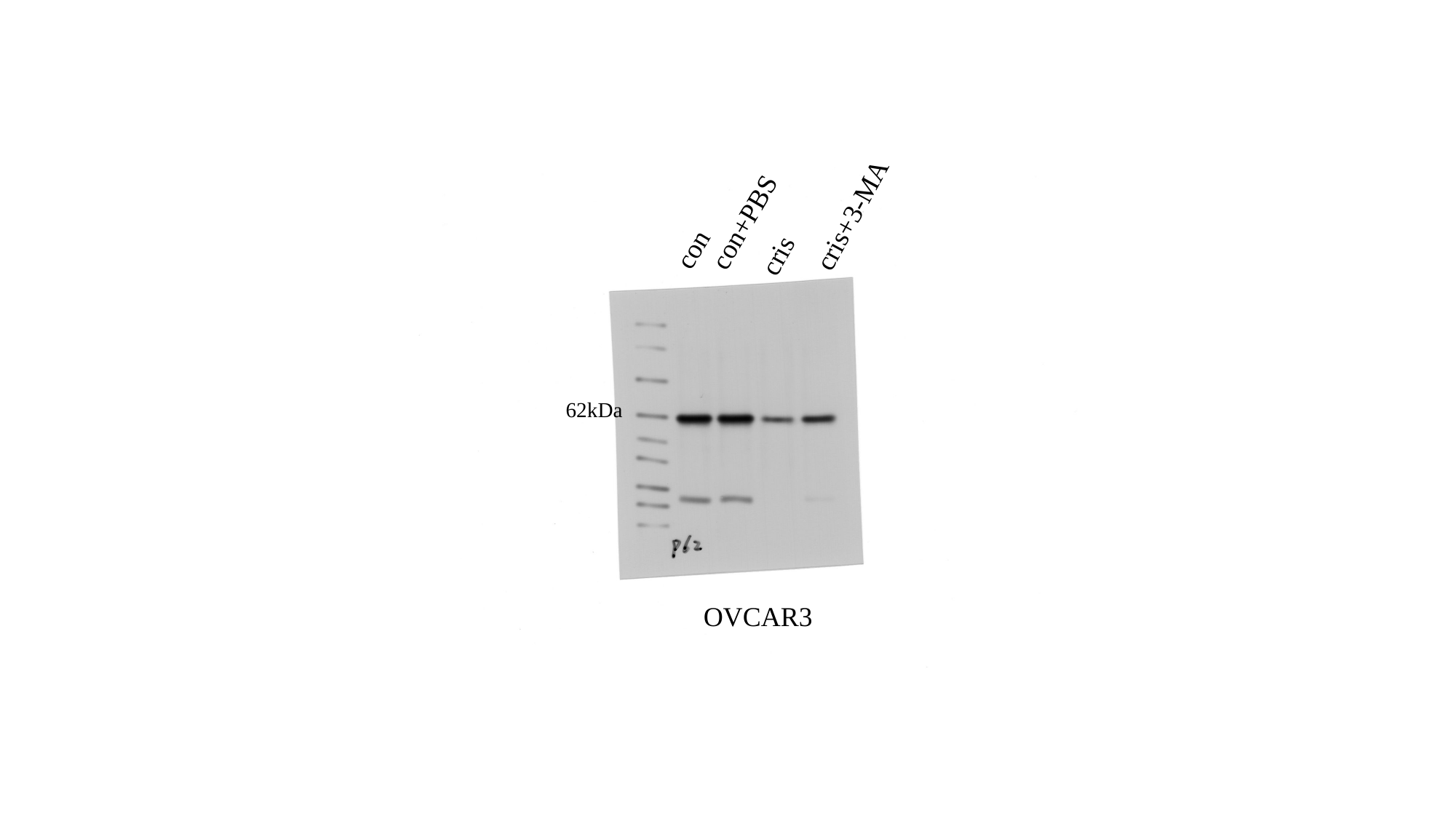

cris+3-MA
con+PBS
con
cris
62kDa
OVCAR3

## Slide 17
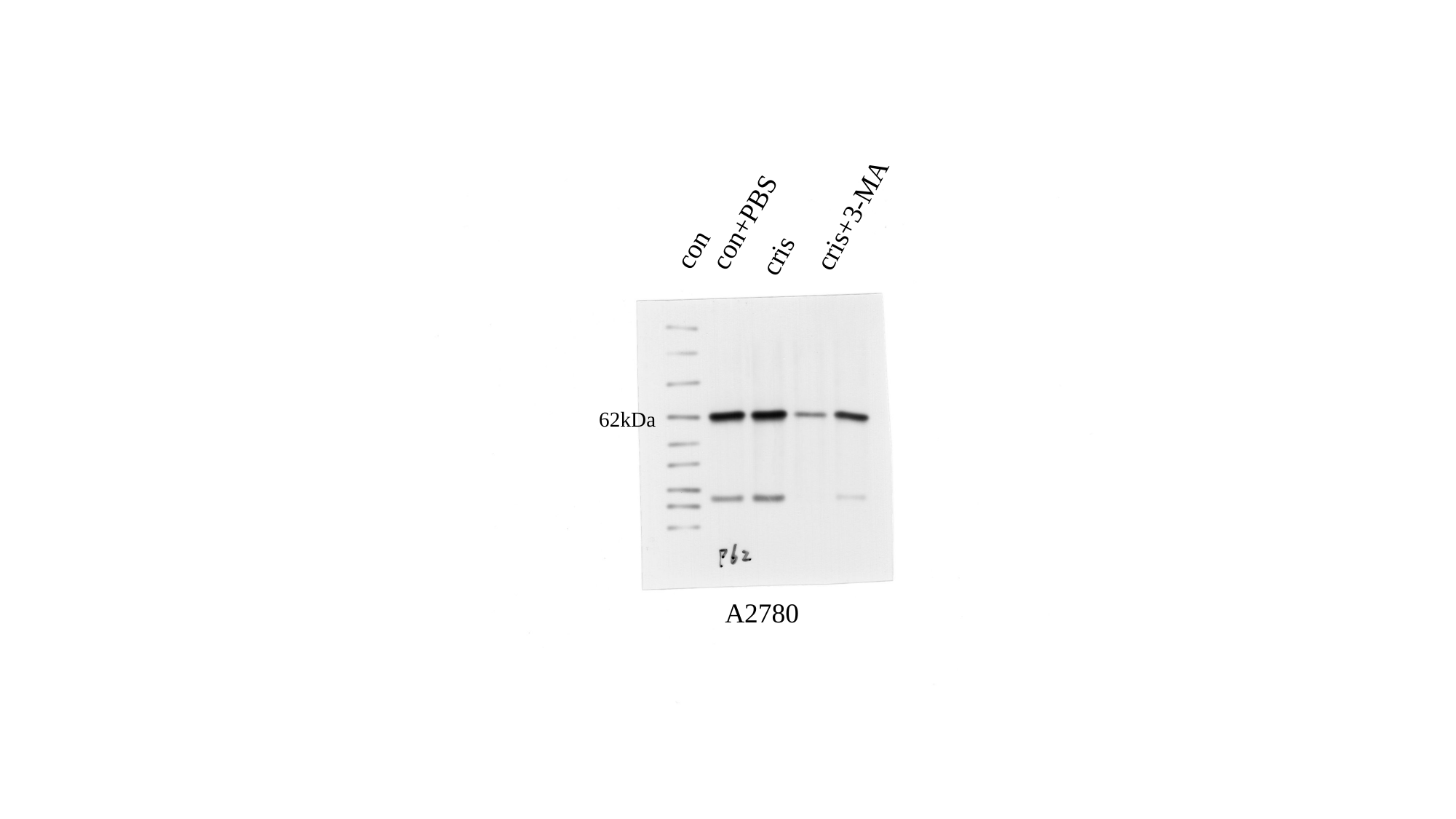

cris+3-MA
con+PBS
con
cris
62kDa
A2780

## Slide 18
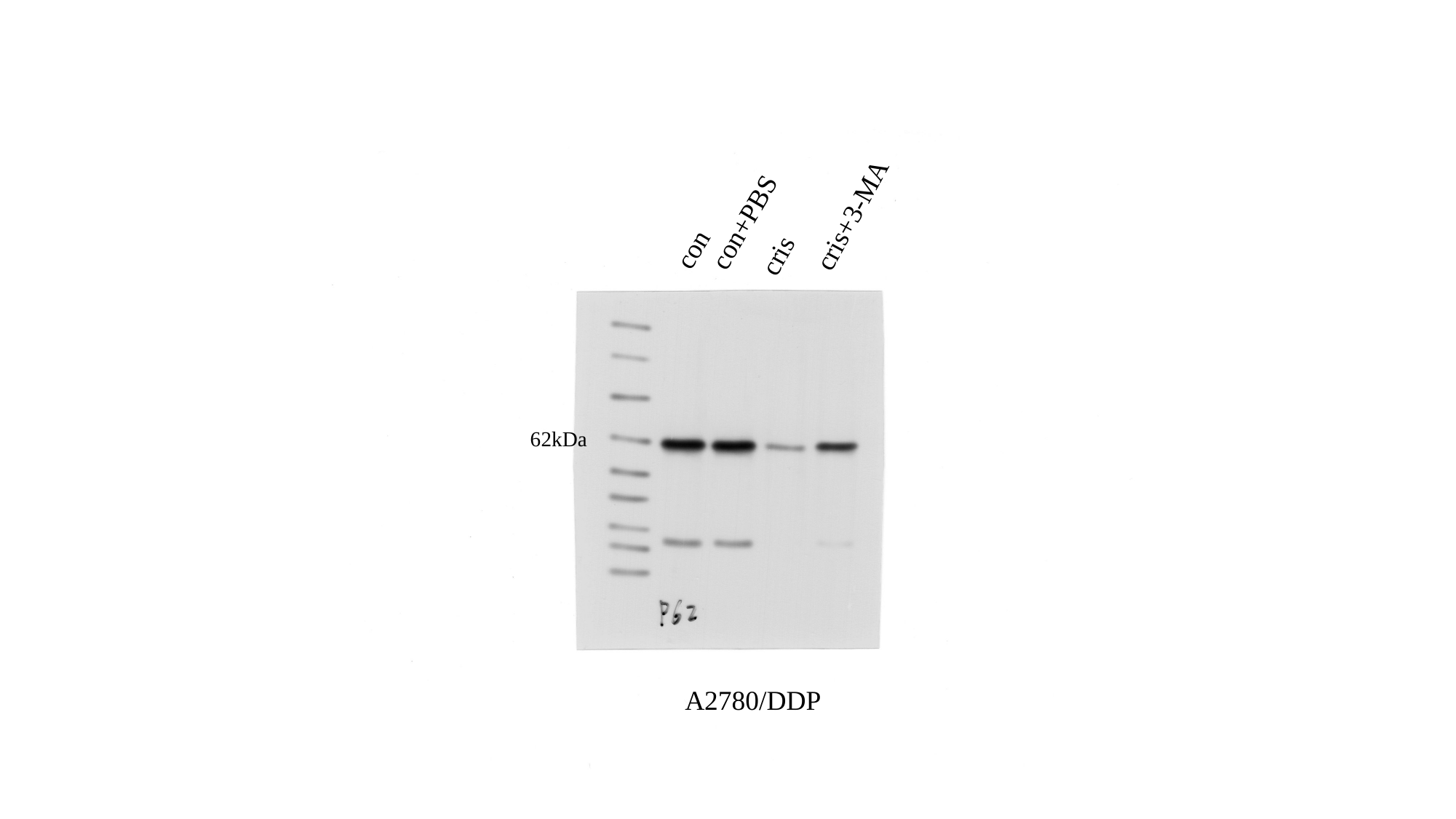

cris+3-MA
con+PBS
con
cris
62kDa
A2780/DDP

## Slide 19
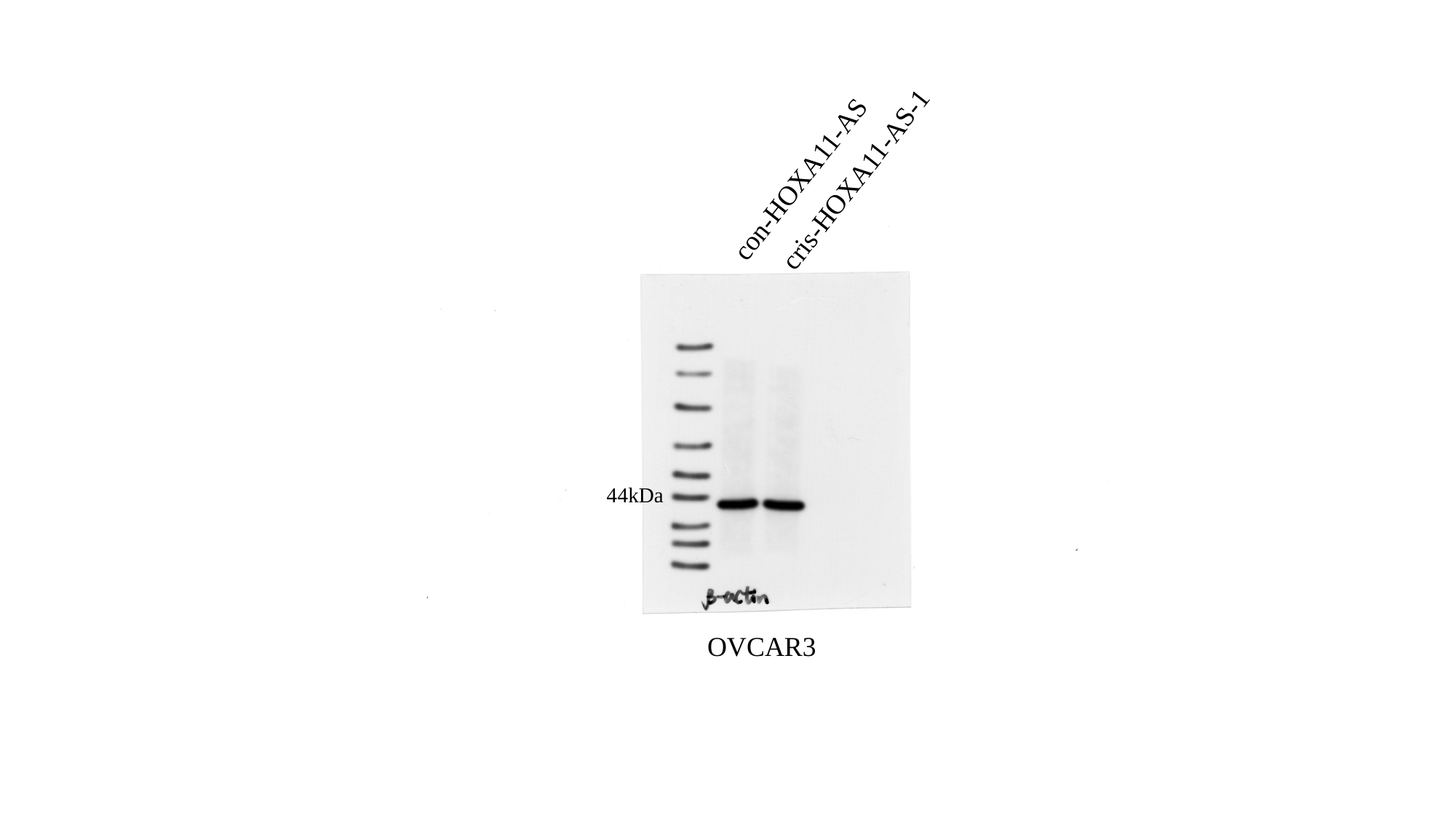

con-HOXA11-AS
cris-HOXA11-AS-1
44kDa
OVCAR3

## Slide 20
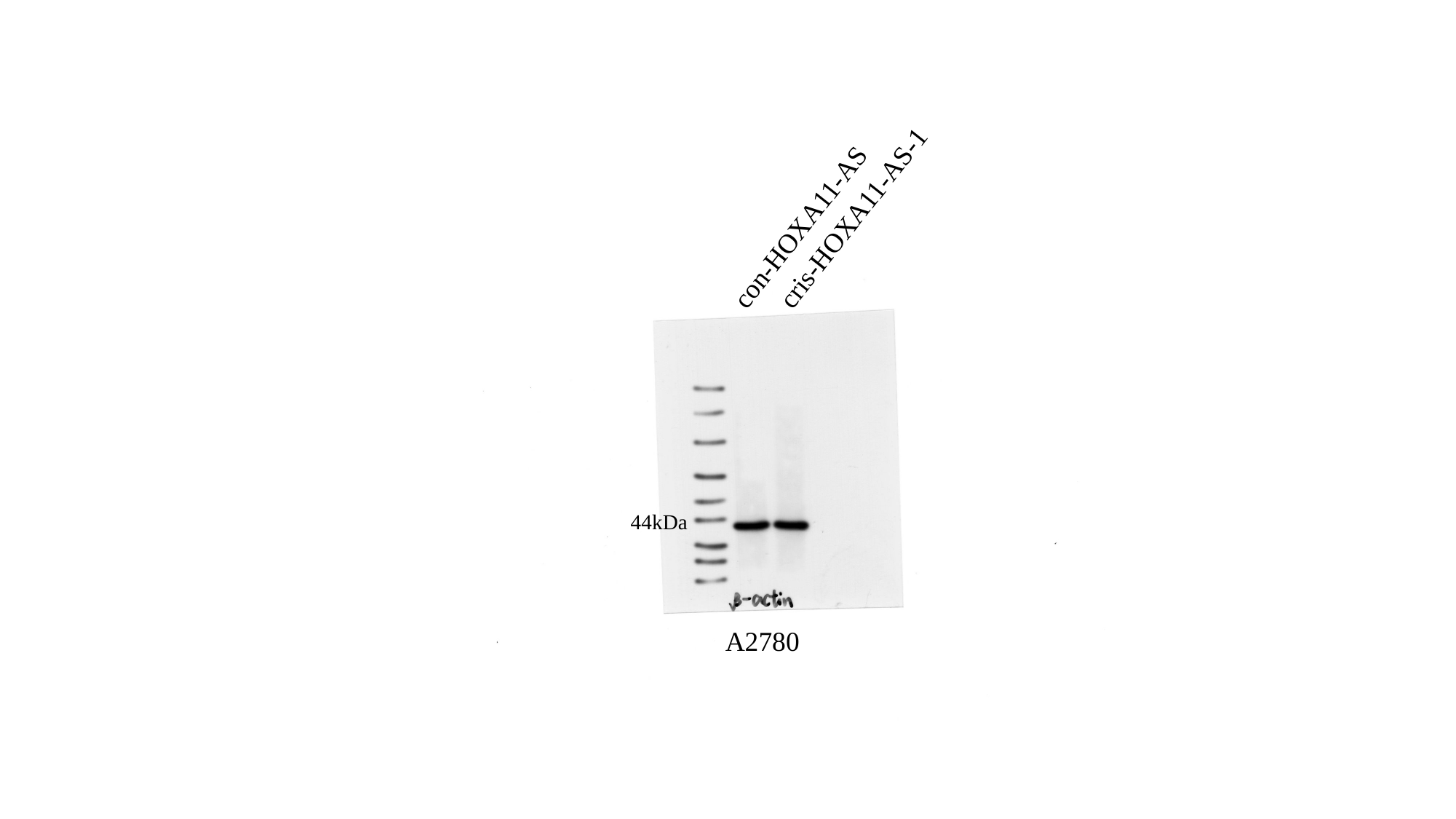

cris-HOXA11-AS-1
con-HOXA11-AS
44kDa
A2780

## Slide 21
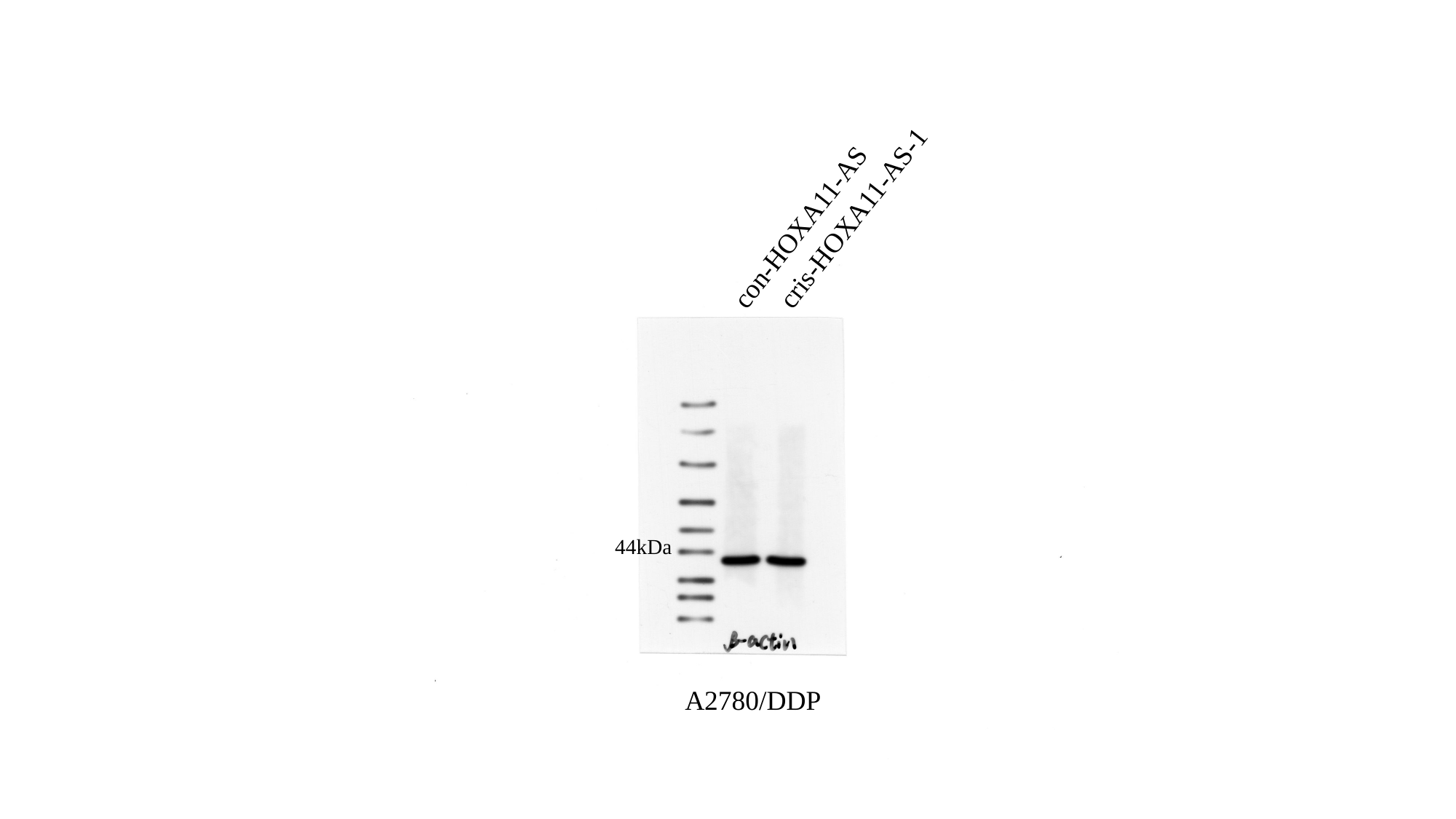

cris-HOXA11-AS-1
con-HOXA11-AS
44kDa
A2780/DDP

## Slide 22
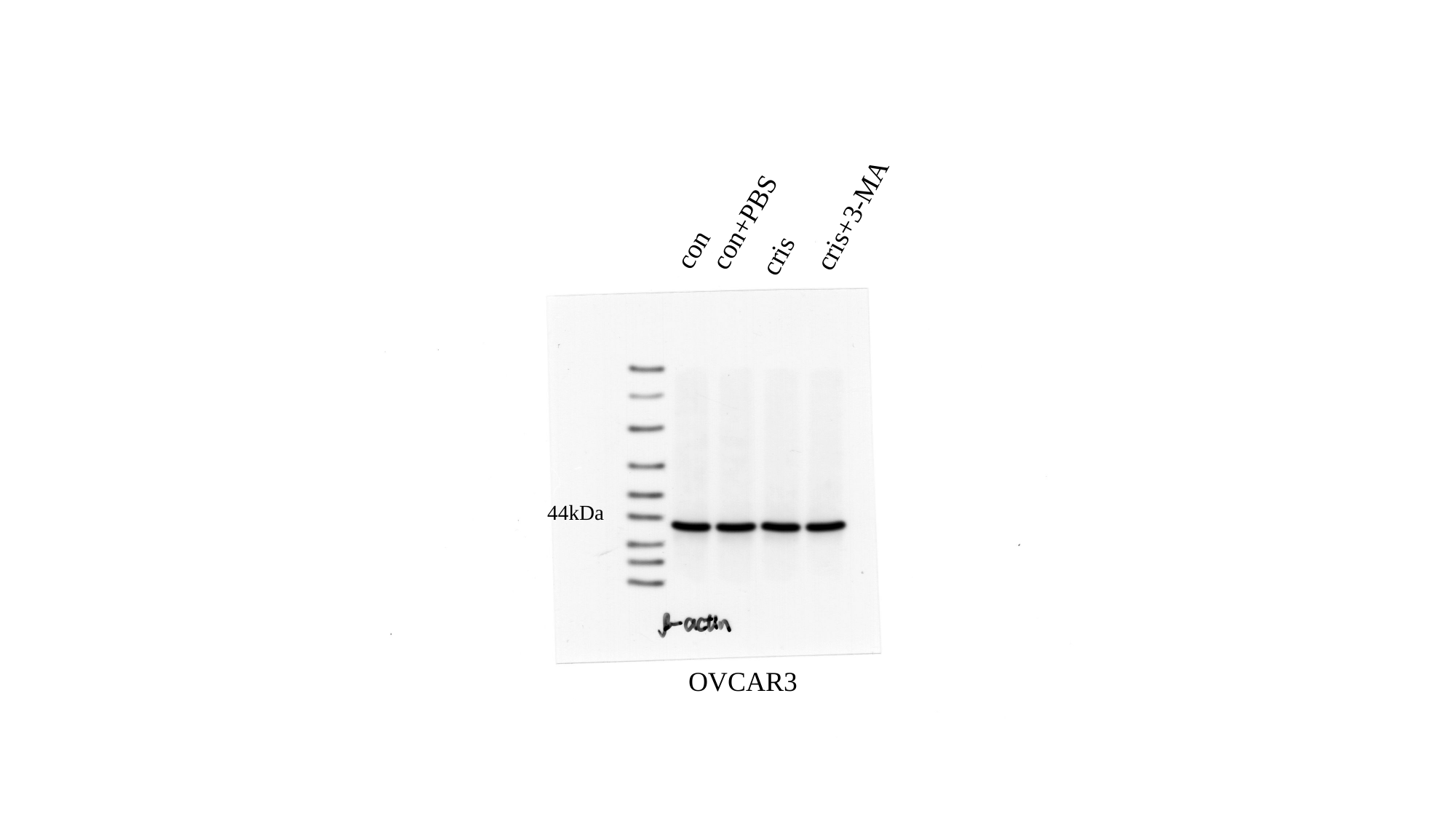

cris+3-MA
con+PBS
con
cris
44kDa
OVCAR3

## Slide 23
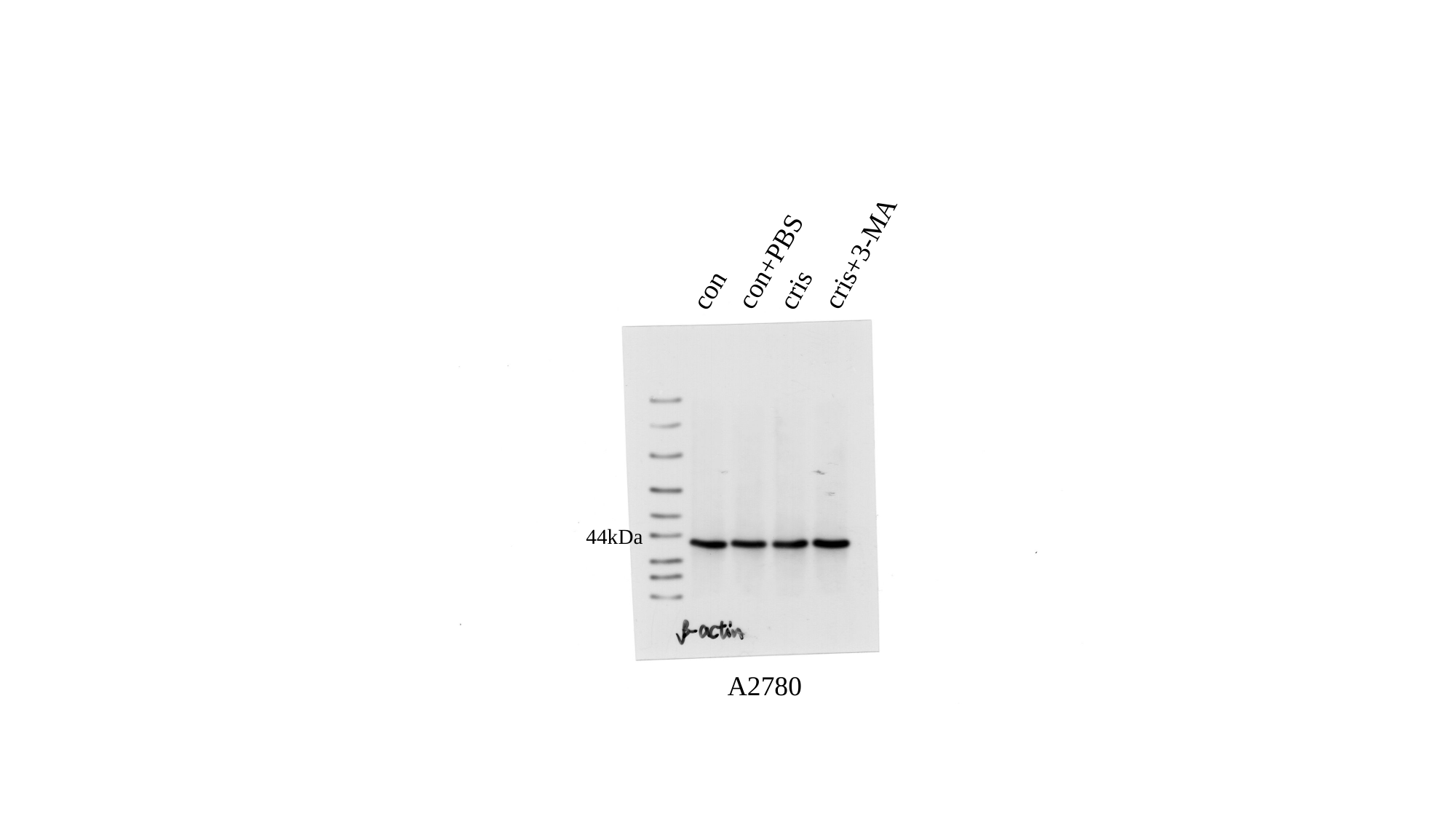

cris+3-MA
con+PBS
cris
con
44kDa
A2780

## Slide 24
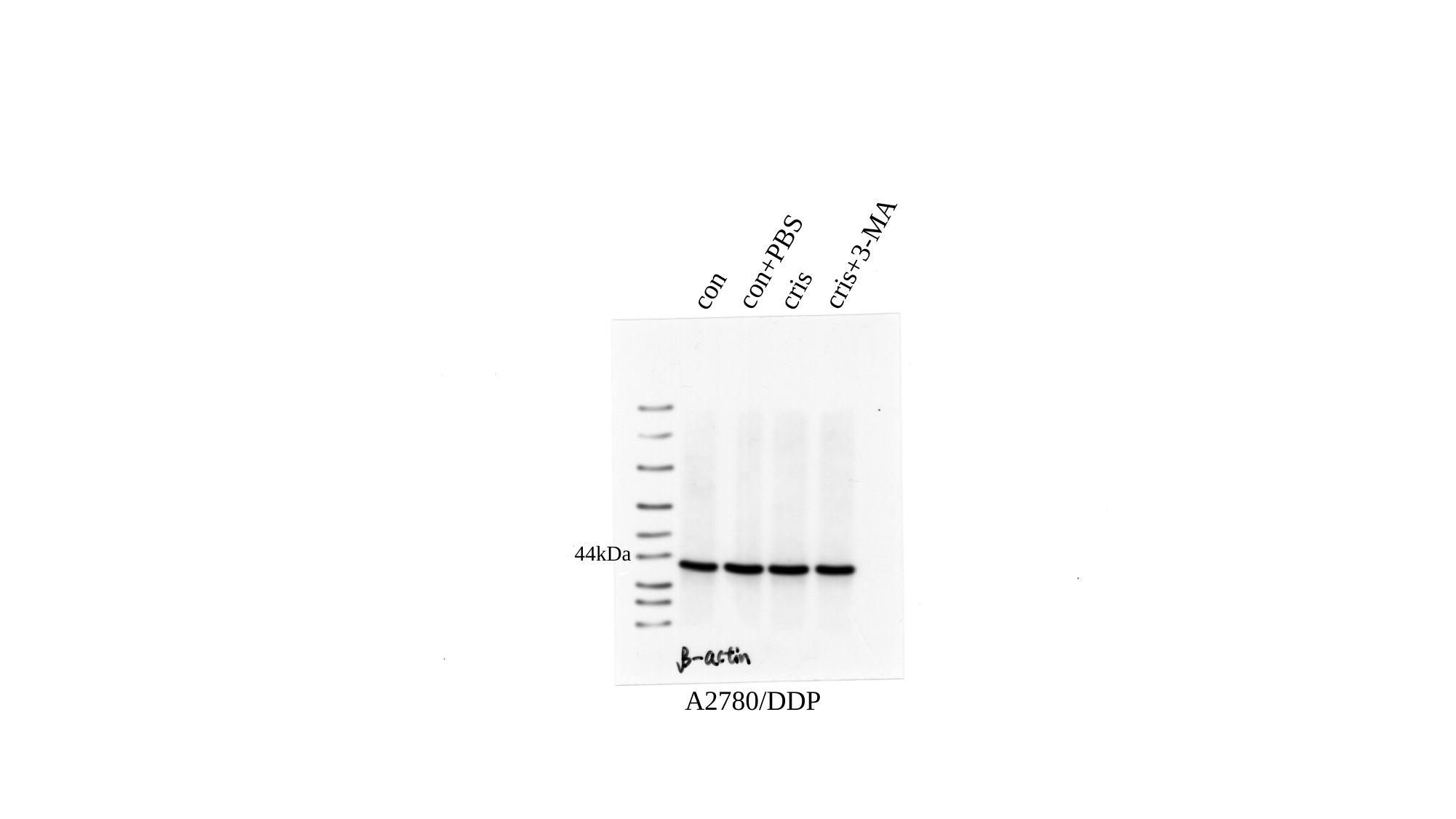

cris+3-MA
con+PBS
cris
con
44kDa
A2780/DDP

## Slide 25
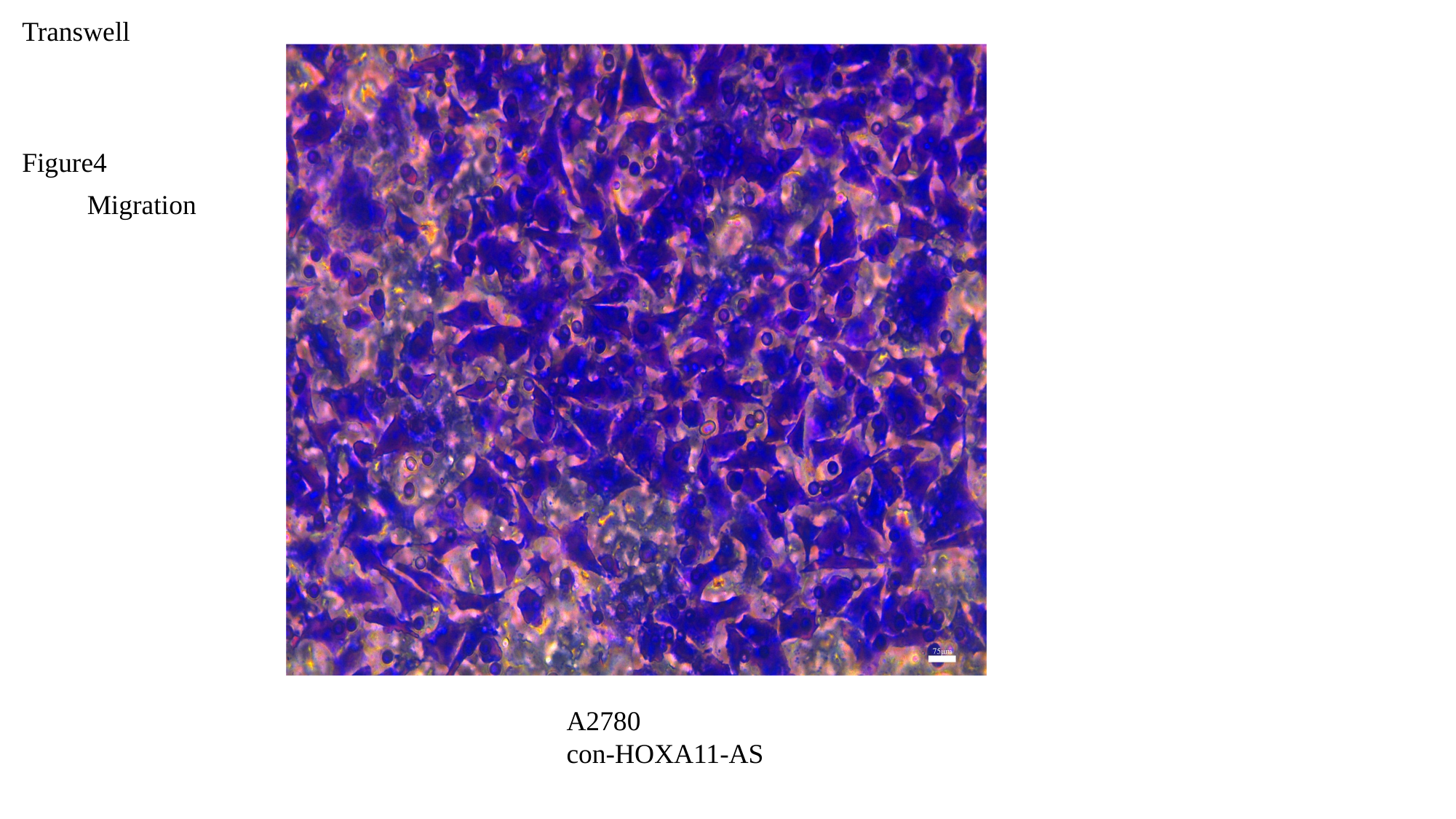

Transwell
Figure4
Migration
A2780
con-HOXA11-AS

## Slide 26
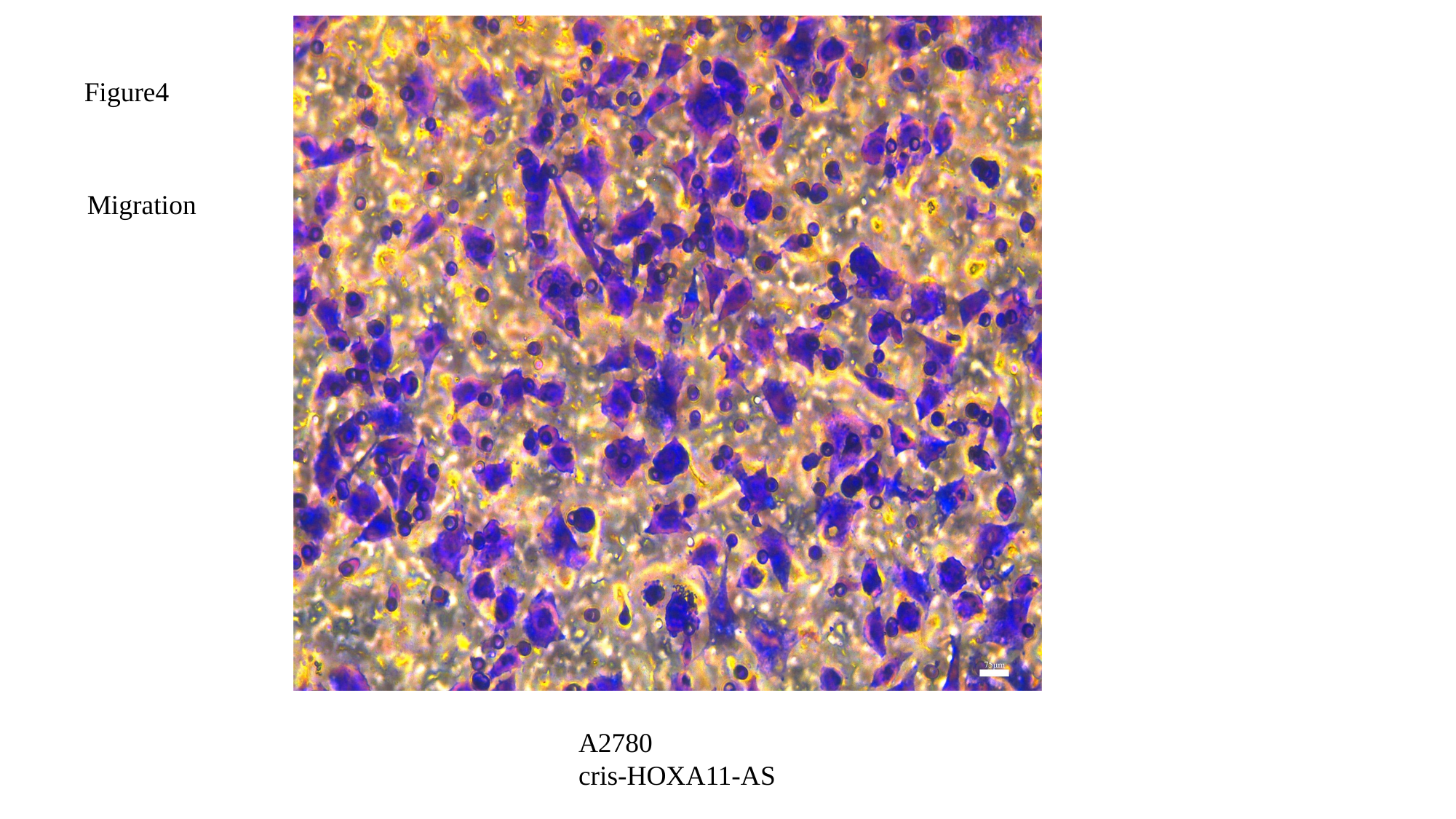

Figure4
Migration
A2780
cris-HOXA11-AS

## Slide 27
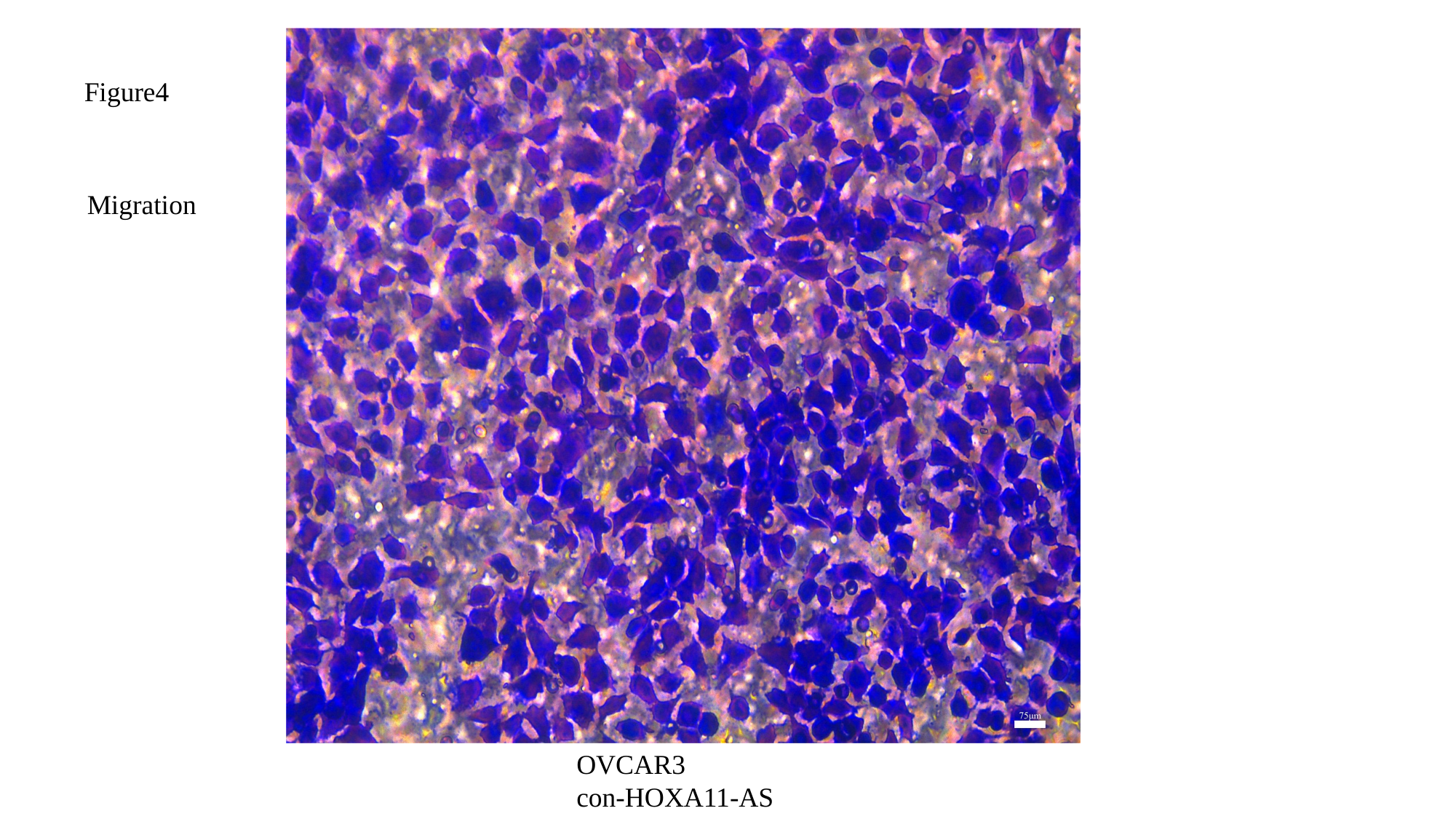

Figure4
Migration
OVCAR3
con-HOXA11-AS

## Slide 28
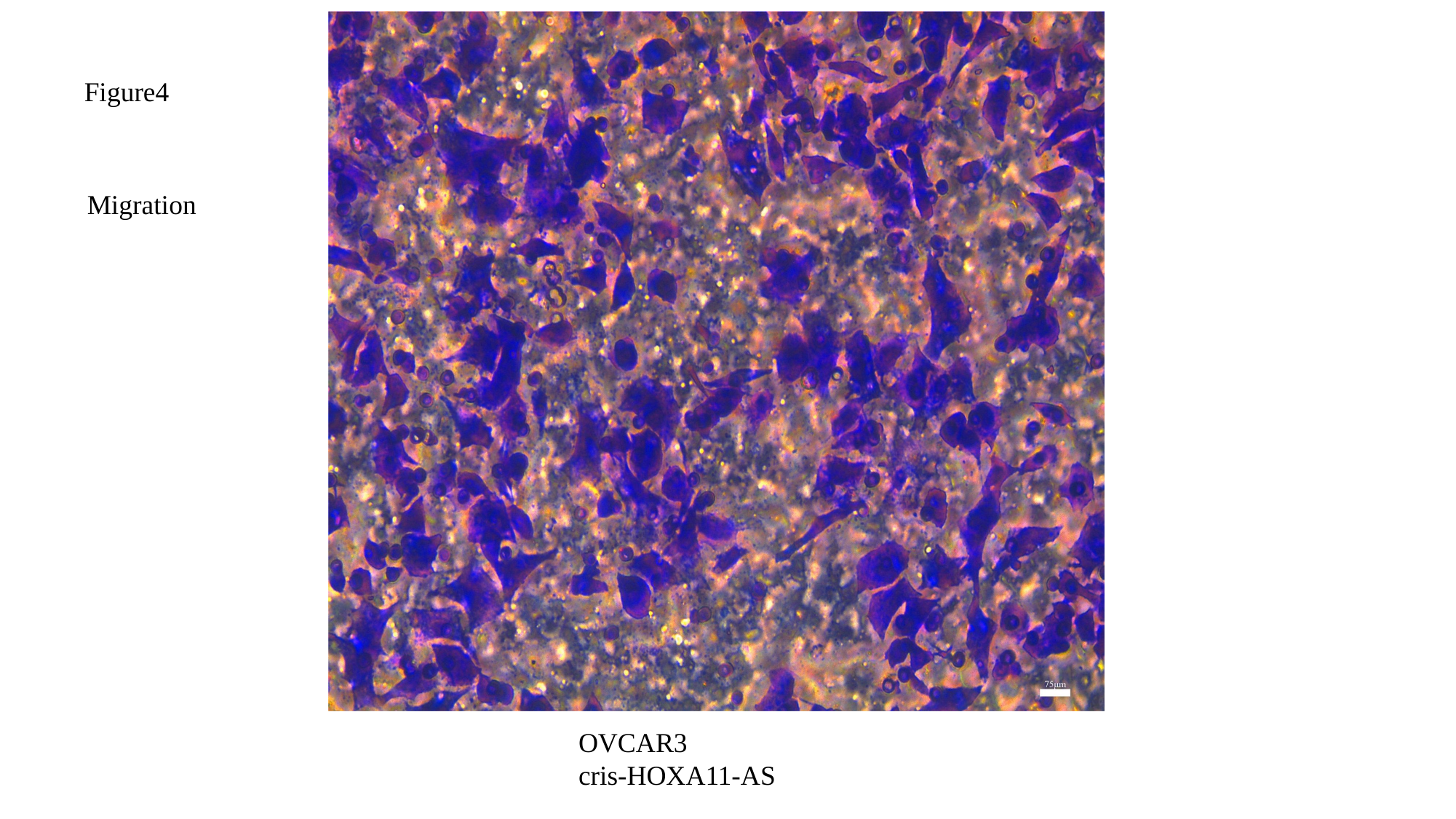

Figure4
Migration
OVCAR3
cris-HOXA11-AS

## Slide 29
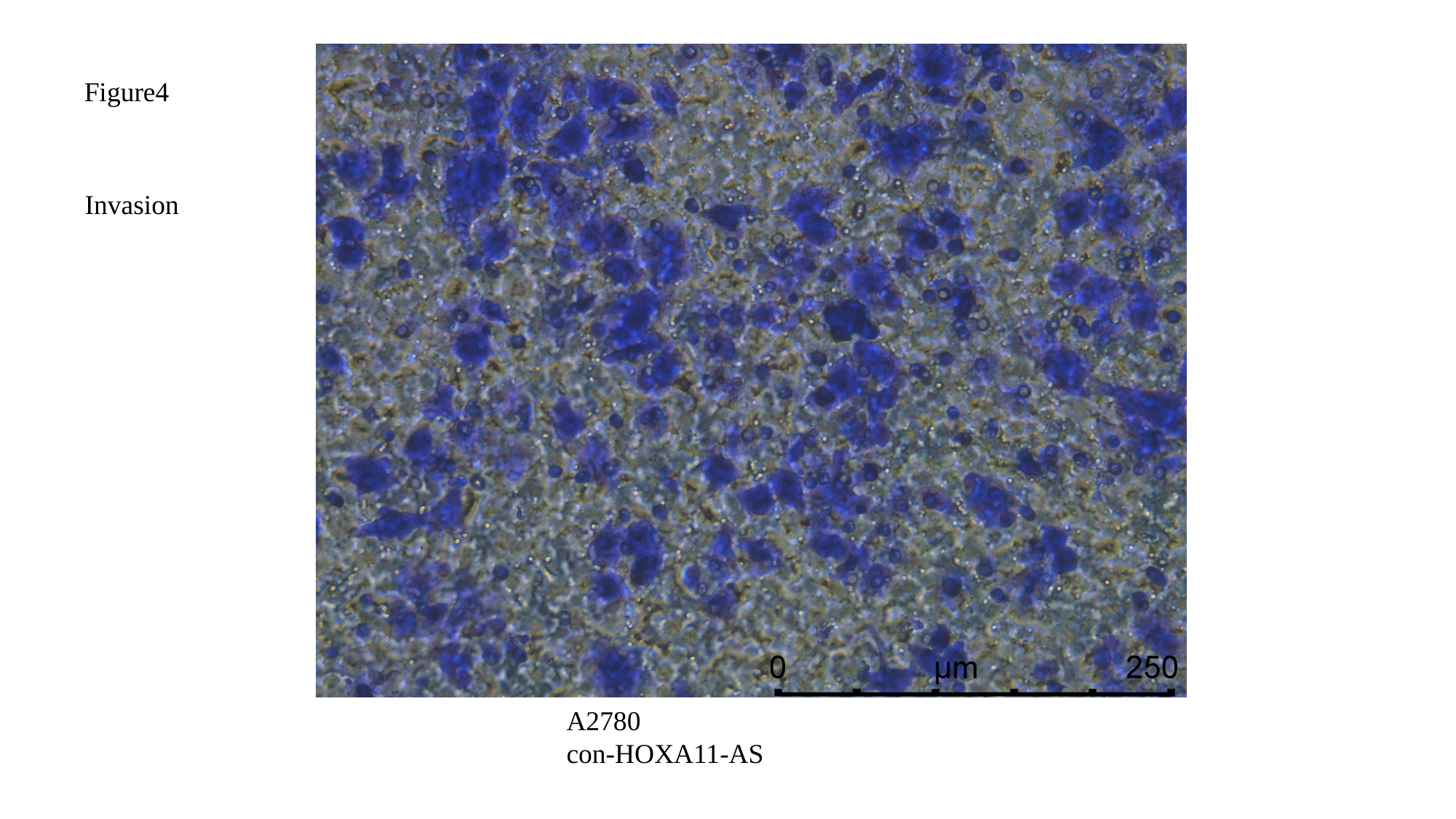

Figure4
Invasion
A2780
con-HOXA11-AS

## Slide 30
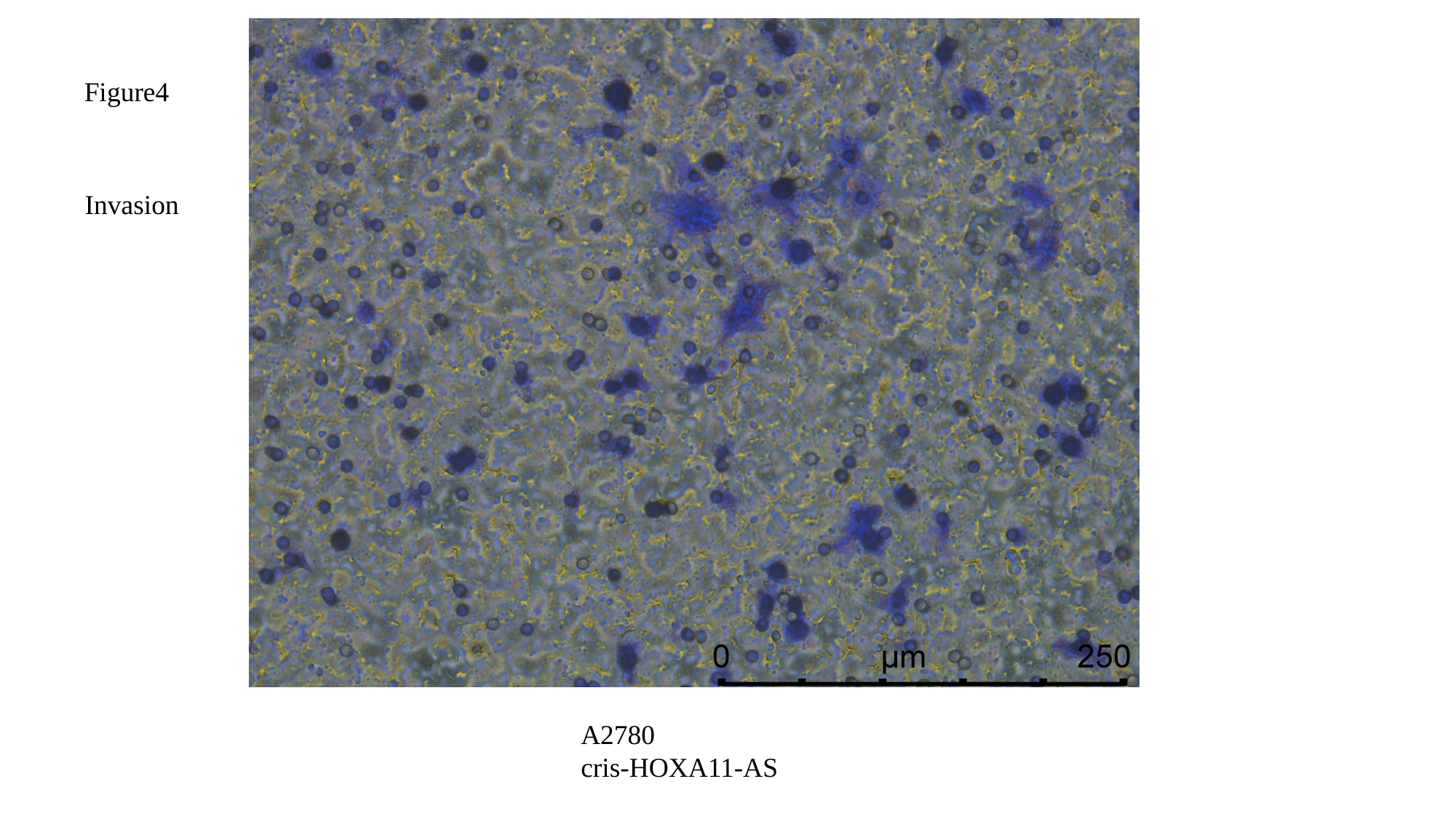

Figure4
Invasion
A2780
cris-HOXA11-AS

## Slide 31
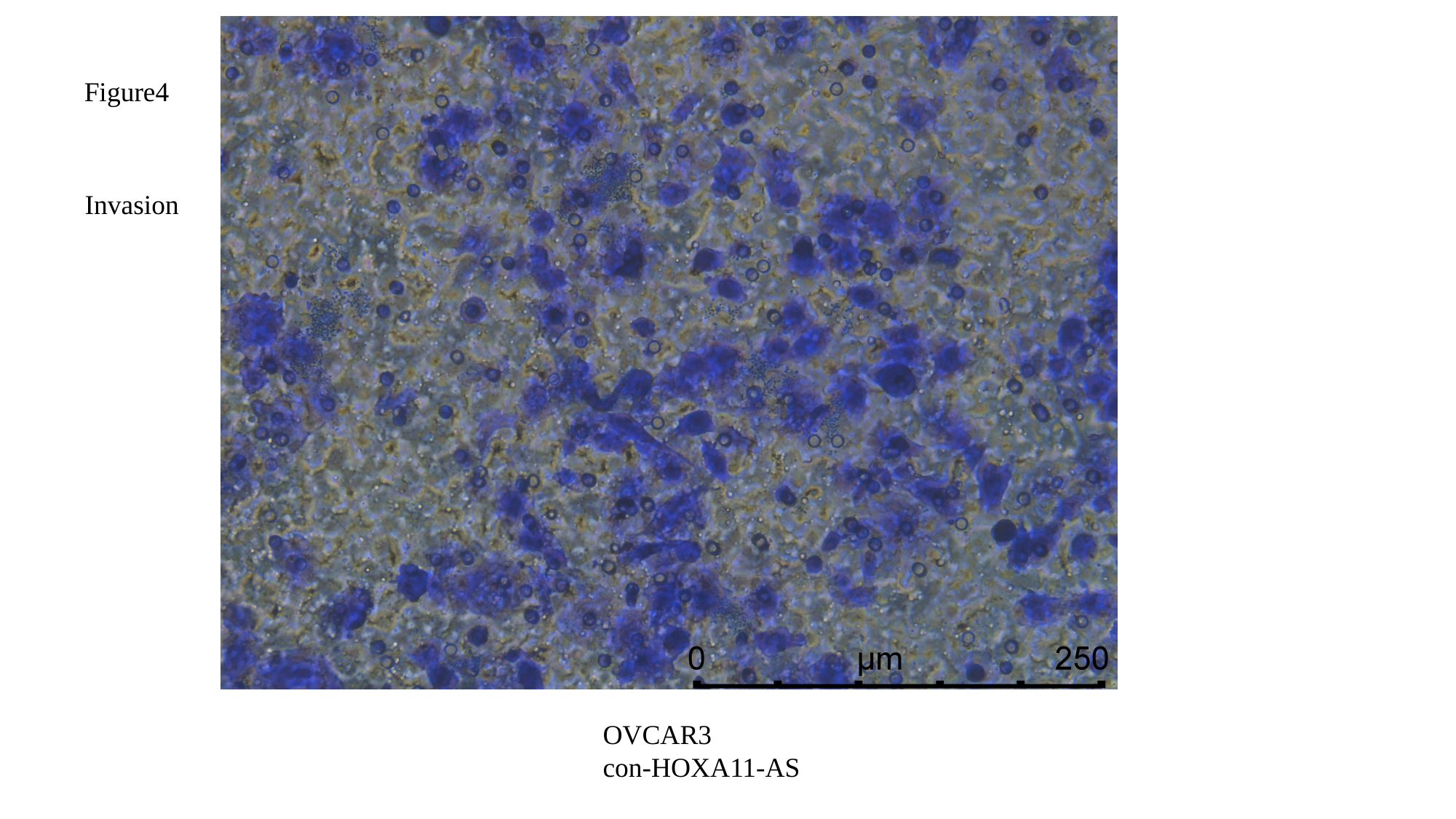

Figure4
Invasion
OVCAR3
con-HOXA11-AS

## Slide 32
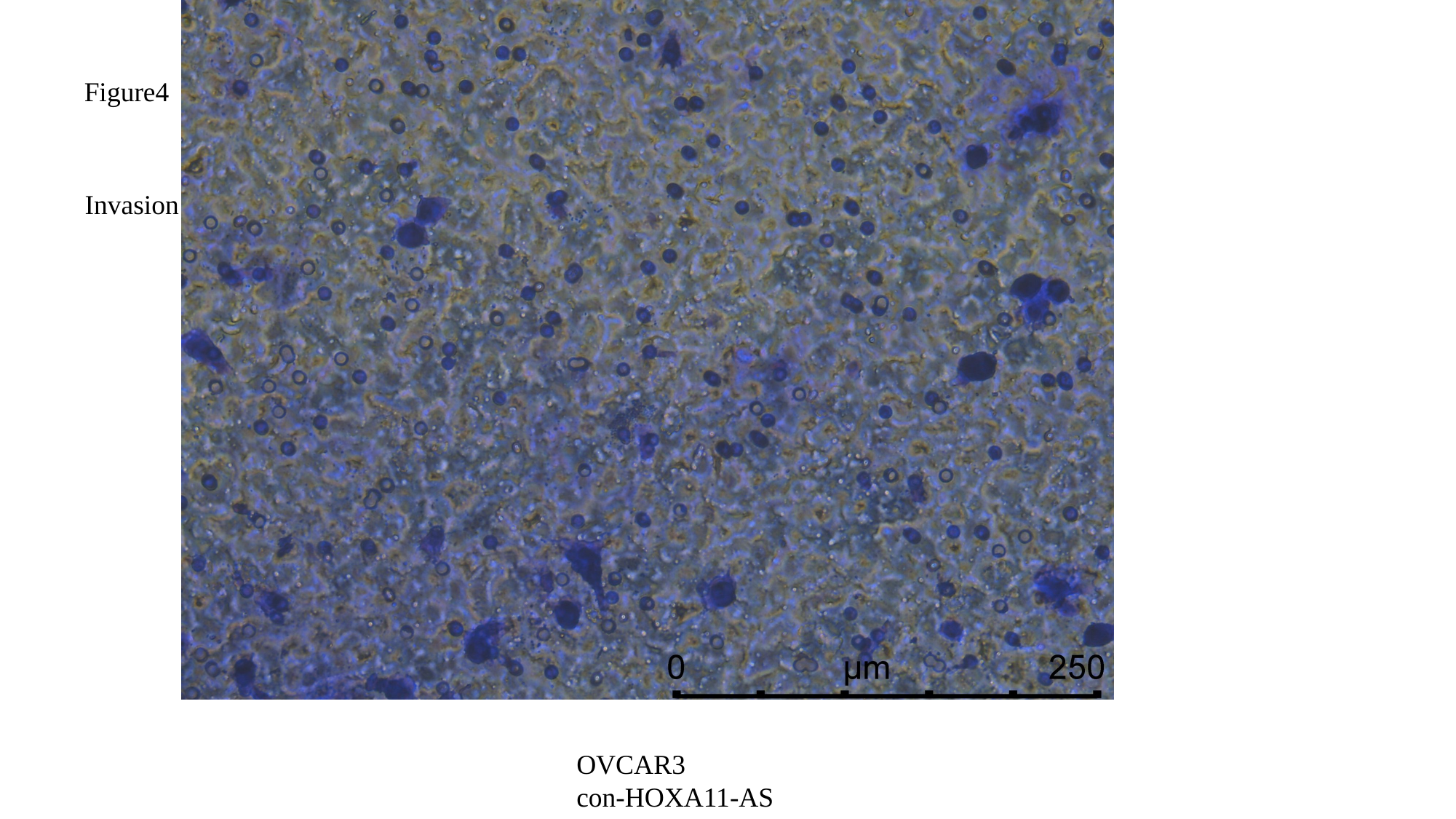

Figure4
Invasion
OVCAR3
con-HOXA11-AS

## Slide 33
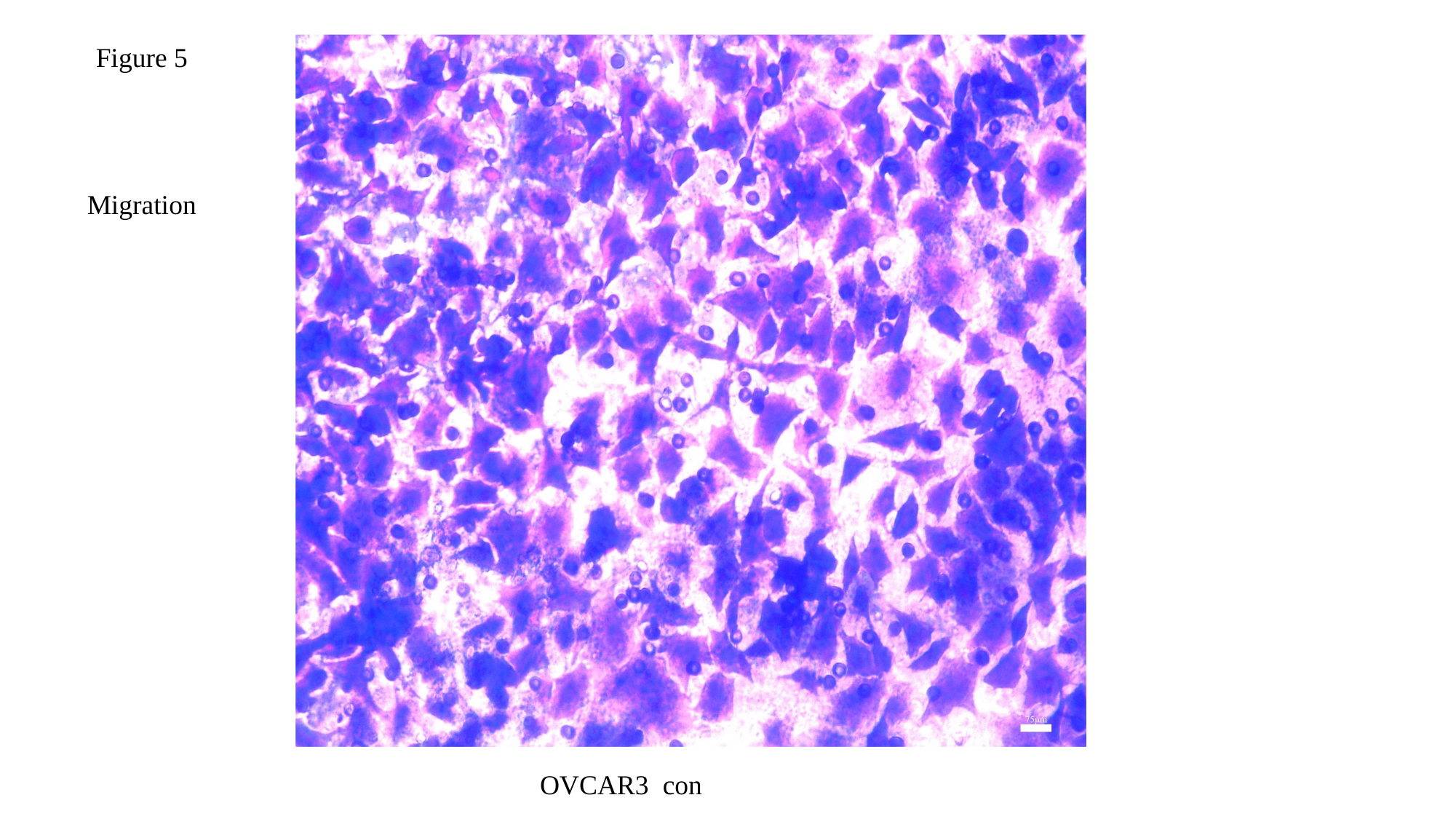

Figure 5
Migration
OVCAR3 con

## Slide 34
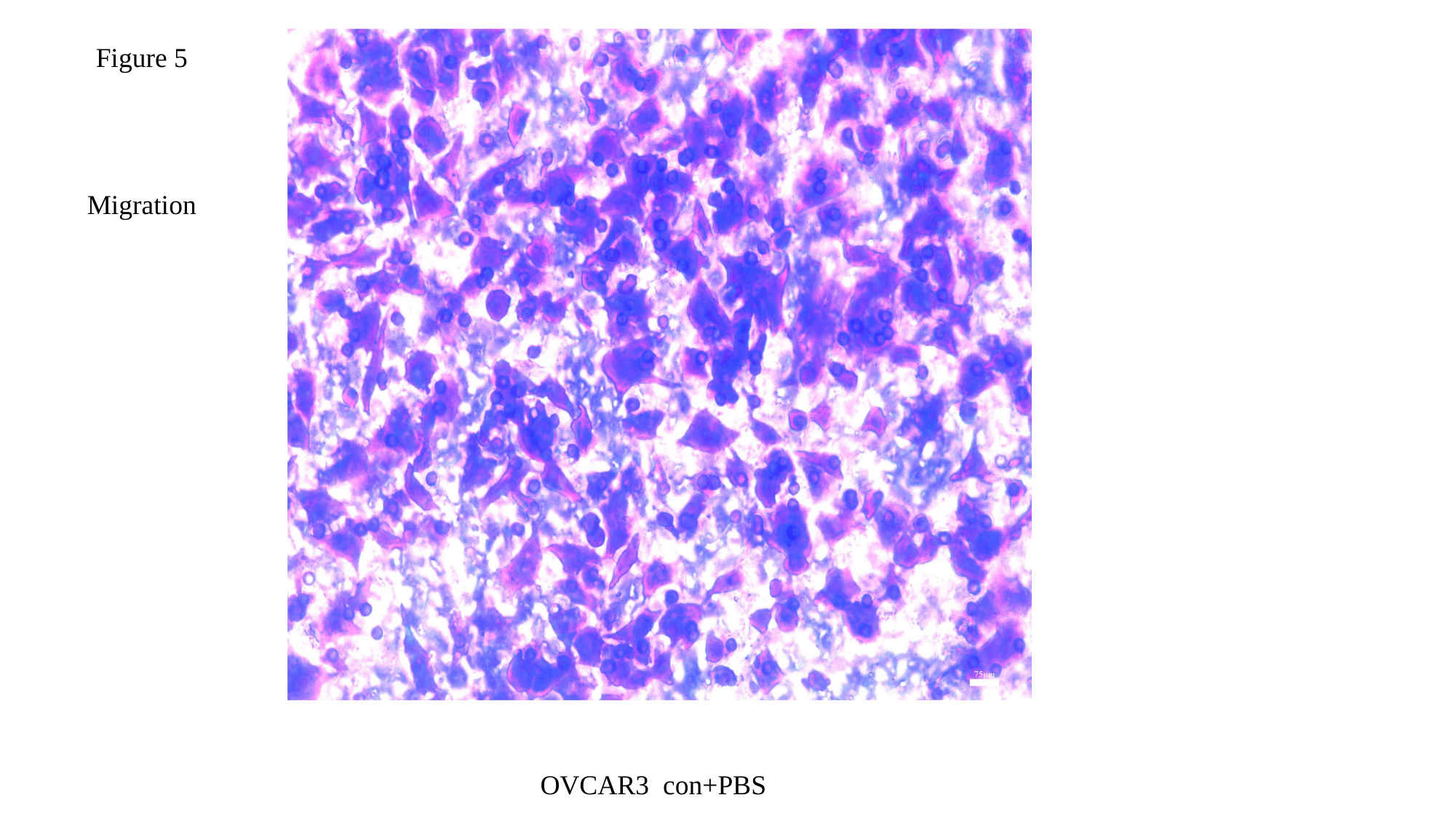

Figure 5
Migration
OVCAR3 con+PBS

## Slide 35
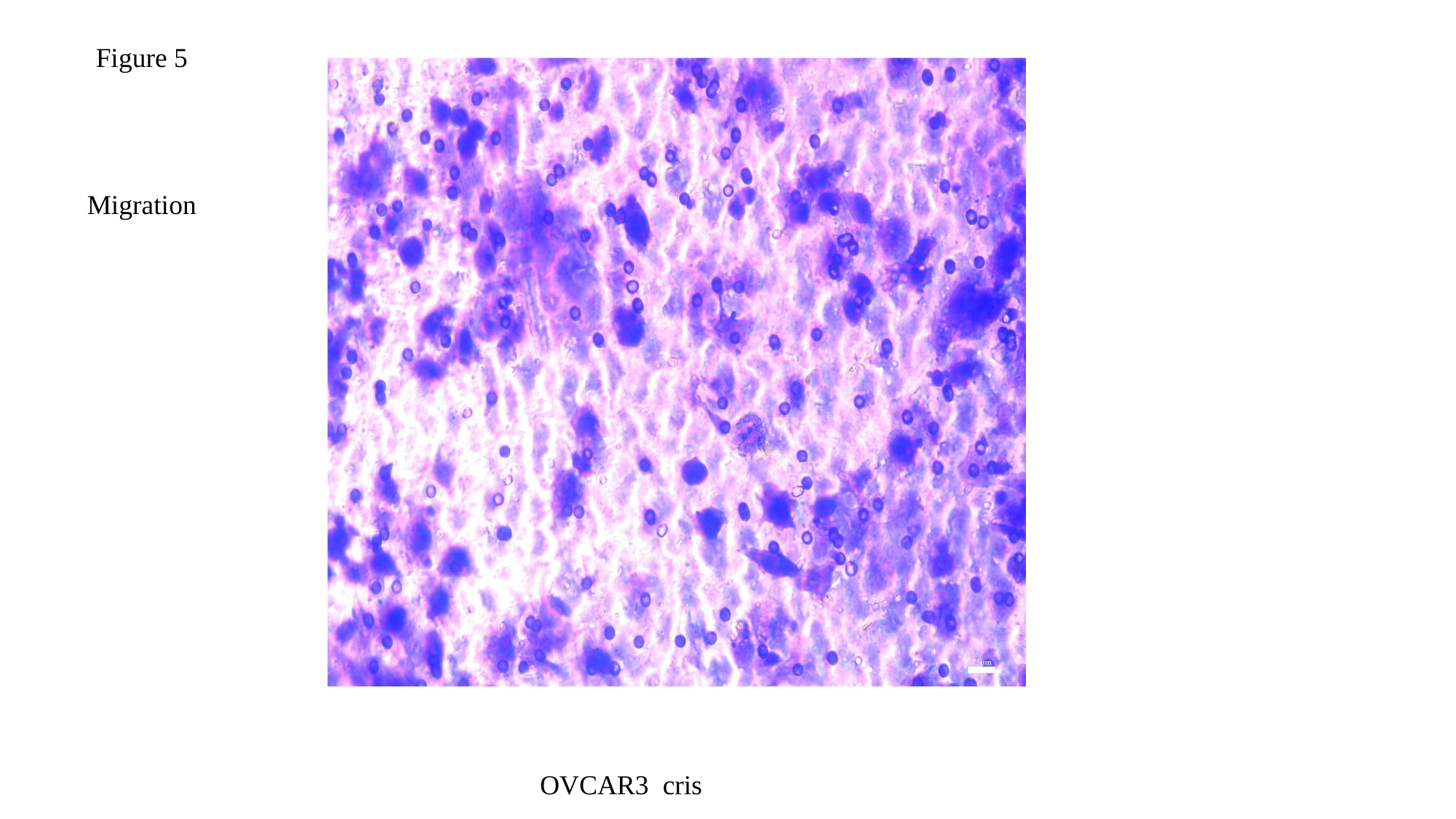

Figure 5
Migration
OVCAR3 cris

## Slide 36
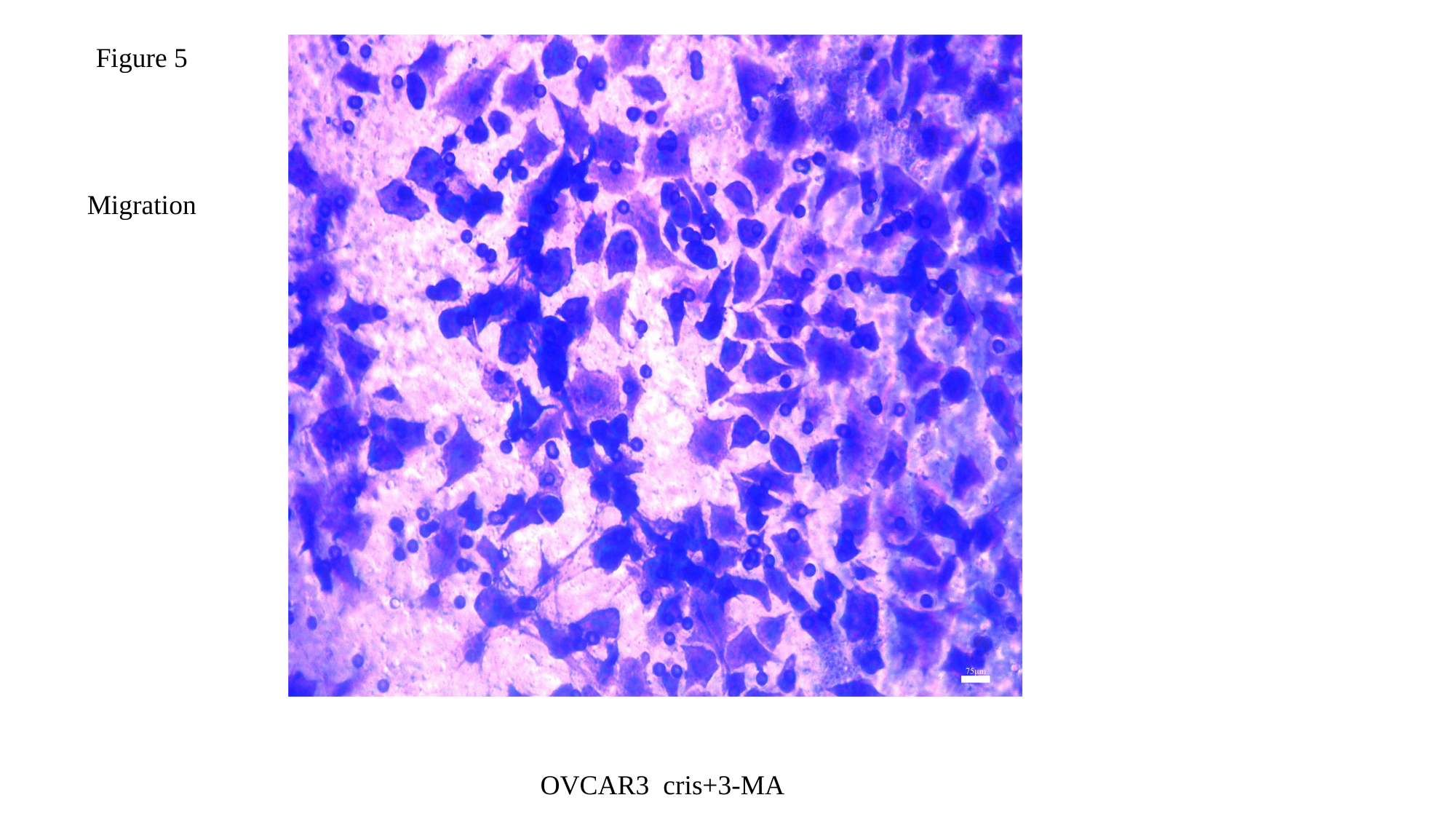

Figure 5
Migration
OVCAR3 cris+3-MA

## Slide 37
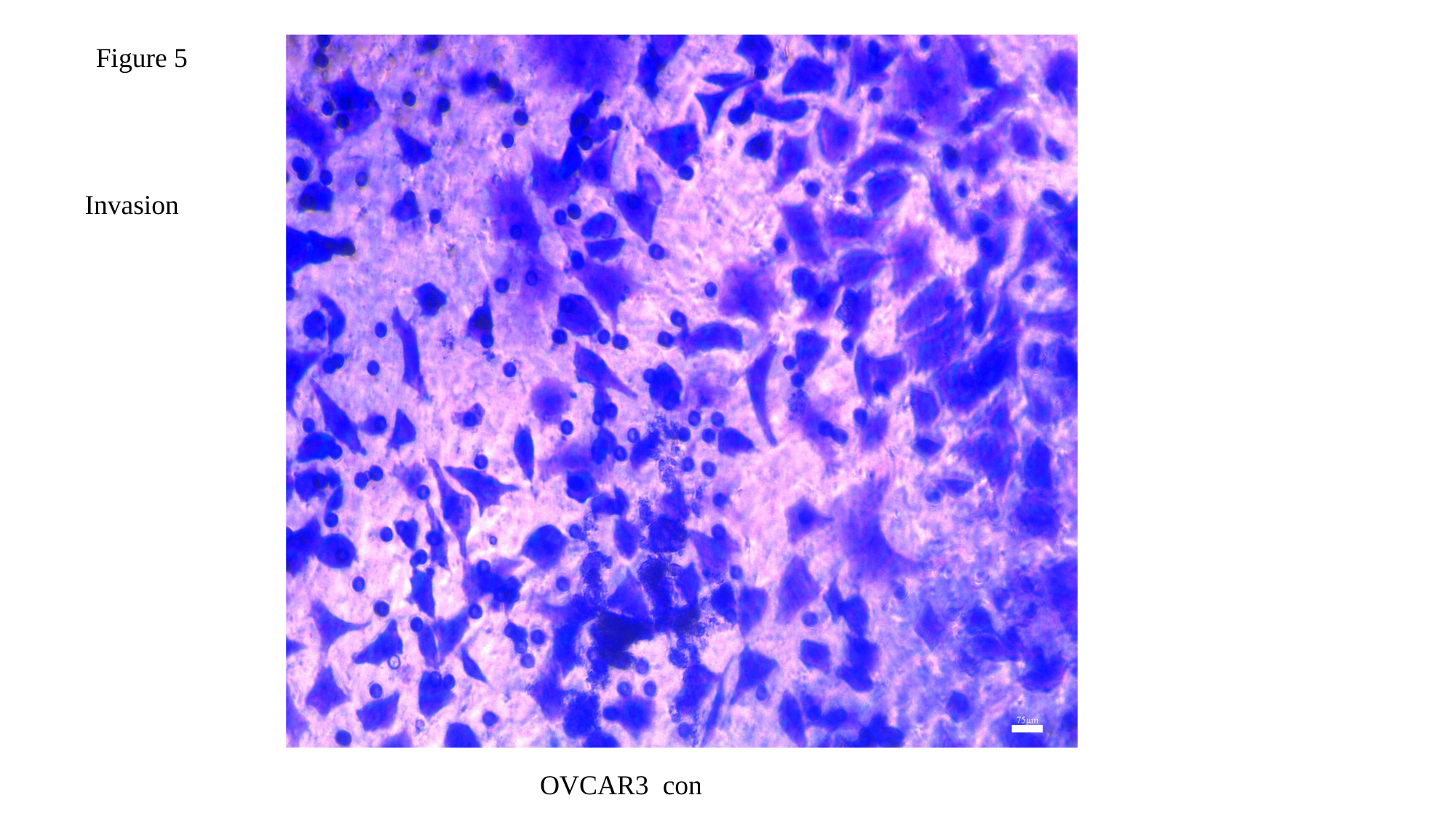

Figure 5
Invasion
OVCAR3 con

## Slide 38
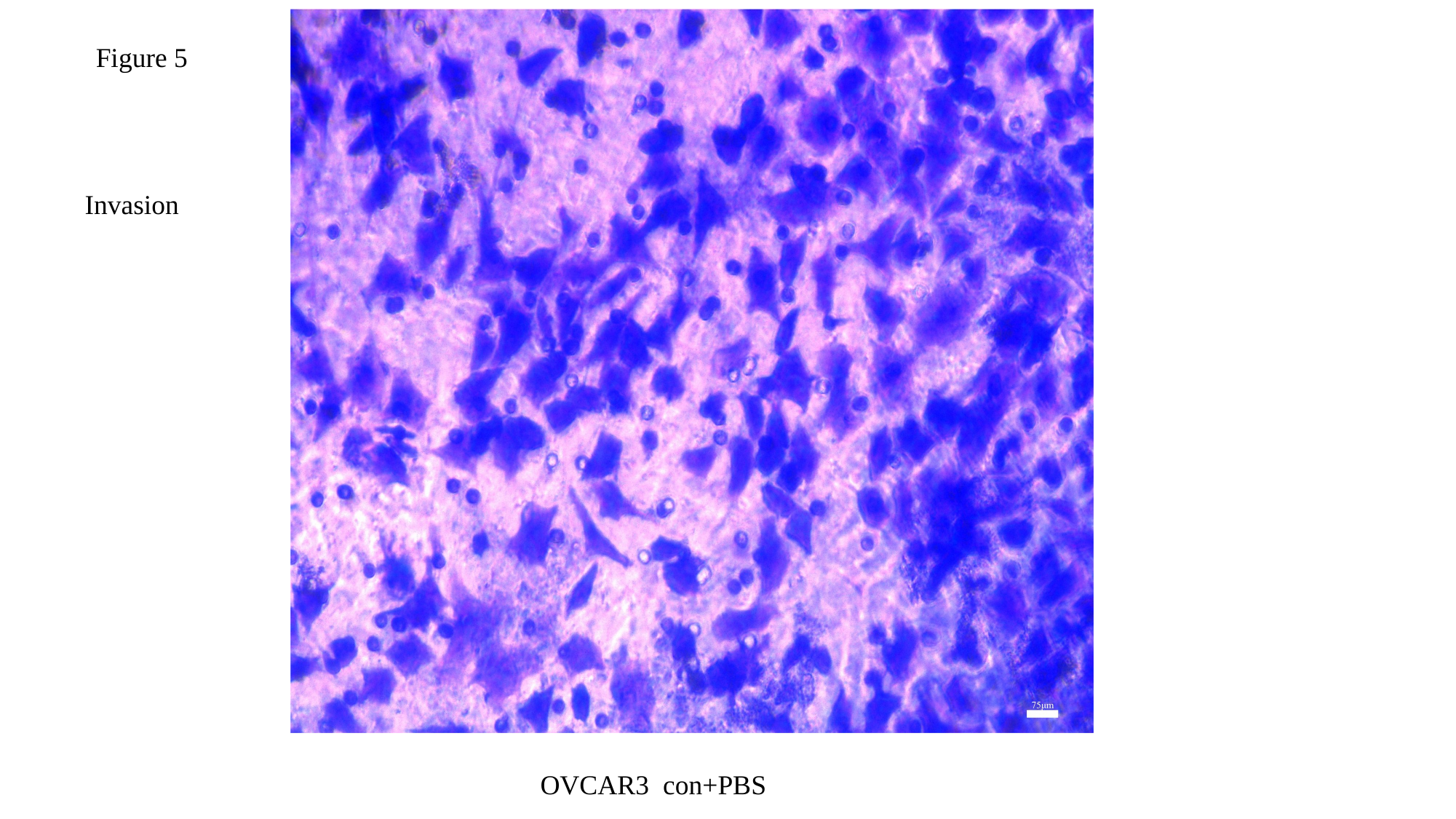

Figure 5
Invasion
OVCAR3 con+PBS

## Slide 39
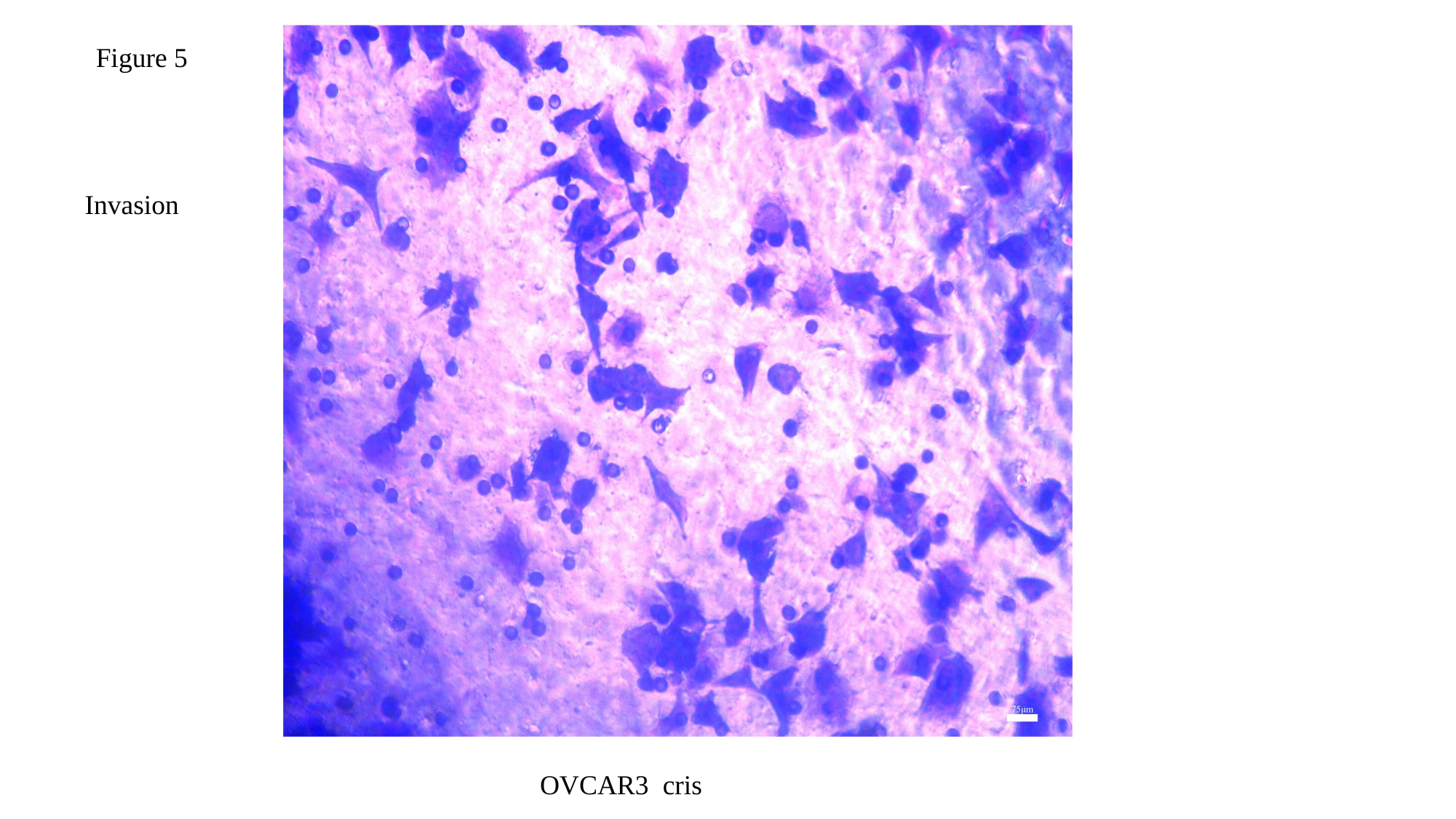

Figure 5
Invasion
OVCAR3 cris

## Slide 40
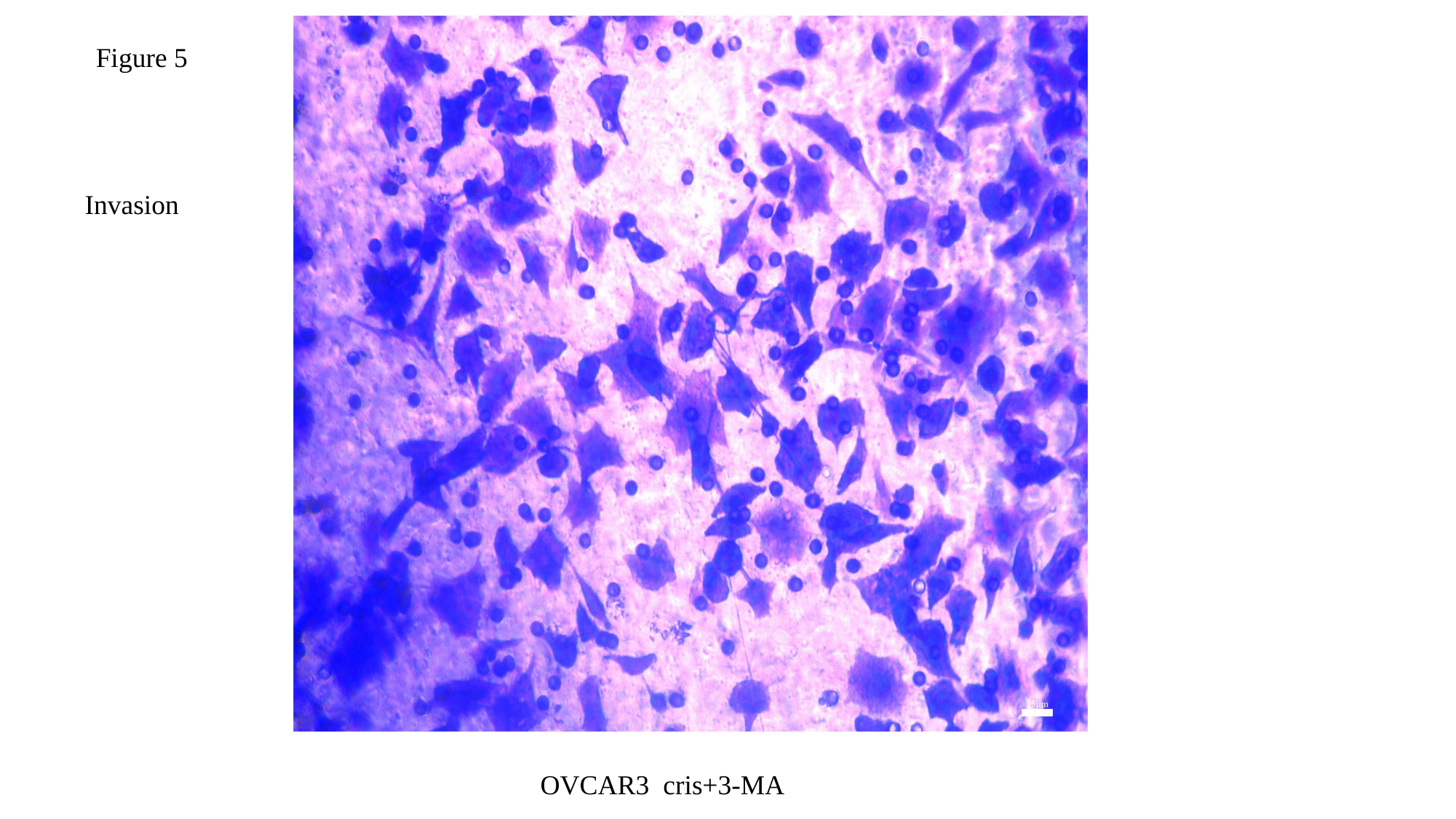

Figure 5
Invasion
OVCAR3 cris+3-MA
